# Long‐term socioeconomic outcomes in Danish children with moderate‐to‐severe atopic dermatitis

**DOI:** 10.1111/jdv.70085

**Published:** 2025-10-03

**Authors:** Sigrun Alba Johannesdottir Schmidt, Dóra Körmendiné Farkas, Mette S. Deleuran, Christian Vestergaard, Henrik T. Sørensen, Niels Skipper, Sinéad M. Langan

**Affiliations:** ^1^ Department of Clinical Epidemiology and Center for Population Medicine Aarhus University Hospital and Aarhus University Aarhus Denmark; ^2^ Department of Dermatology Aarhus University Hospital Aarhus Denmark; ^3^ Department of Economics and Business Economics Aarhus University Aarhus Denmark; ^4^ Faculty of Epidemiology and Population Health London School of Hygiene and Tropical Medicine London UK; ^5^ Health Data Research UK

**Keywords:** atopic dermatitis, cohort studies, Denmark, employment, epidemiology, family, income, registries, sick leave, socioeconomic factors

## Abstract

**Background:**

The long‐term socioeconomic impact of atopic dermatitis (AD) is poorly understood.

**Objectives:**

To examine if childhood AD is associated with labor market and relationship outcomes in adulthood.

**Methods:**

This nationwide registry‐based Danish cohort study included children born between 1973 and 1991: 8409 with hospital‐diagnosed AD before age 18 (baseline) and 853,228 without. A sibling cohort (5119 with AD; 6352 without) was used to control for family‐related confounding. The main outcomes were earned income, long‐term unemployment, single partnership status and childlessness by age 30. We used linear regression for income and Poisson regression for binary outcomes, adjusted for income, sex, calendar year, comorbidities and childhood socioeconomic status.

**Results:**

Children with AD had slightly lower income (adjusted mean percentile difference − 1.2) and higher risk of long‐term unemployment (adjusted relative risk [aRR] 1.11; 95% CI: 1.05–1.17) by age 30, though the absolute difference was <1%. AD was not associated with secondary labor market outcomes (e.g. health‐related work absenteeism), except for disability pension, but the RR decreased from 1.55 to 1.15 (95% CI: 1.02–1.30) when adjusting for comorbidities. By age 30, 22.1% with AD remained single versus 19.4% without (aRR 1.11; 95% CI: 1.06–1.15), with stronger associations among males, those with severe eczema, hand/contact dermatitis or low maternal education (aRRs up to 1.41). AD was not associated with childlessness at age 30 (57.7% vs. 56.7%; aRR 1.01; 95% CI: 1.00–1.03), but a 2.5% absolute difference appeared by age 40. In the sibling analysis, most associations diminished.

**Conclusions:**

Moderate‐to‐severe childhood AD was not linked to labor market outcomes, apart from disability pensions, likely driven by comorbidities. The probability of partnership or parenthood was reduced in specific subgroups. Confounding from family‐related factors cannot be excluded. Nevertheless, the results underscore the importance of considering patients' broader life situations to support them through adulthood.

## Plain Language Summary

Atopic dermatitis (also known as eczema) is a common skin disease that often begins in childhood. It is characterized by dry, itchy, and inflamed skin and can affect both health and quality of life. While its short‐term effects are well known, the long‐term impact on people’s work and relationships is unclear.

Using nationwide Danish registries, we identified 8,409 children with and 853,228 children without a hospital diagnosis of eczema before age 18 and compared their socioeconomic outcomes at age 30. We also compared patients with their siblings to account for family‐related factors.

As adults, individuals with childhood eczema had a similar income and risk of long‐term unemployment compared with those without eczema. However, they were more likely to receive disability pensions, especially if they also had other health problems. In their personal relationships, they were slightly more likely to remain single up to age 30, especially men, those with more severe disease, and those from families with fewer resources. The likelihood of becoming a parent by age 30 was not substantially affected, although small differences emerged later in life. Differences were smaller when comparing siblings with and without eczema.

This study suggests that although childhood eczema does not generally impact work outcomes in adulthood, it may have lasting effects on relationships. Support for patients should therefore address not only skin symptoms but also broader life challenges.

## Graphical abstract

This cohort study using Danish registry data found that children with hospital‐diagnosed atopic dermatitis (AD) had slightly lower income and a higher risk of long‐term unemployment by age 30. Certain subgroups had a reduced likelihood of forming relationships and becoming parents. Most associations weakened in sibling comparisons, indicating family‐related confounding.

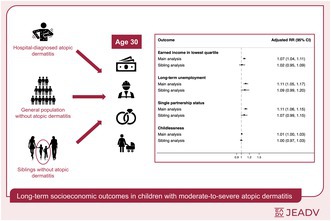

**Why was the study undertaken?**

Atopic dermatitis (AD) affects both health and psychosocial well‐being, but its long‐term socioeconomic impact is poorly understood. This study examined if childhood hospital‐diagnosed atopic dermatitis influences adult labor market and relationship outcomes.

**What does this study add?**

In this Danish registry‐based cohort study, children with moderate‐to‐severe AD had similar adult income and employment to their peers. However, they were more likely to receive disability pensions (especially with comorbidities), and certain subgroups (e.g. those with severe eczema) were less likely to find a partner or become a parent. Associations were weaker when comparing patients with their siblings.

**What are the implications of this study for disease understanding and/or clinical care?**

Childhood AD may have lasting effects on social and relationship domains. Holistic care should address not only physical health but also the broader life situations to support patients through adulthood.

## INTRODUCTION

Atopic dermatitis (AD), affecting 20% of children, is characterized by itching eczematous lesions.^1^ AD affects both physical health and psychosocial functioning.^2^ Reduced sleep quality due to itching can cause daytime sleepiness and loss of productivity at school and work.^2^ Stigmatization accompanying visible lesions can further influence participation in school and extracurricular activities and cause social isolation.^2^, ^3^, ^4^, ^5^ Interpersonal strain may affect romantic relationships through sexual health impairment and concerns about procreation, for example because of heritability.^2^, ^6^

These challenges might negatively affect labor market outcomes (e.g. health‐related absence and hiring) and the likelihood of finding a partner and becoming a parent. Recognizing such effects is crucial to identify individuals for whom early interventions in childhood might support optimal life‐cycle skill formation and decrease social disparities later in life.^7^

However, population‐based studies investigating long‐term socioeconomic repercussions of childhood AD compared with healthy peers are limited and have methodological shortcomings.^8^, ^9^, ^10^, ^11^, ^12^ Existing evidence has come primarily from cross‐sectional studies,^13^, ^14^, ^15^, ^16^, ^17^, ^18^, ^19^ which are susceptible to reverse causation because stress from financial difficulties, work, unemployment or relationship issues can exacerbate AD. Additionally, many studies have not accounted for comorbidities and socioeconomic background.^8^, ^9^, ^10^, ^11^, ^12^

We used routinely collected data from nationwide Danish registries to conduct a total population and discordant sibling cohort study examining the hypothesis that AD negatively affects labor market and relationship outcomes, particularly in individuals with severe or early‐onset AD, women and individuals from socioeconomically disadvantaged families.

## METHODS

In Denmark, health and social welfare are supported through tax‐financed provision of universal healthcare, education and social services, for example student aid, disability pensions and unemployment insurance.^20^ Detailed data on provided services are recorded in nationwide registries using unique personal identifiers enabling individual‐level registry linkage. The Supplement describes the study's detailed conceptual framework (Appendix S1), data sources (Appendix S2) and coding of variables (Table S1).

### Study population

**FIGURE 1:**
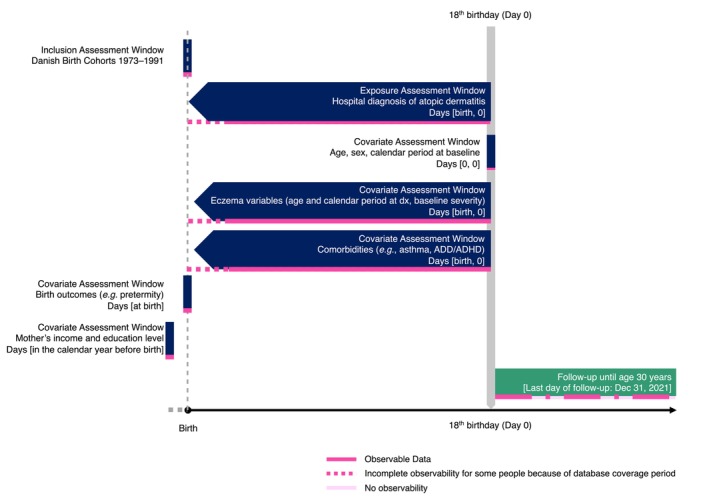
Study design diagram. ^a^Because of the availability of follow‐up data on in vitro fertilization (IVF) treatment until December 31, 2018, 1989–1991 birth cohorts were excluded from the analysis of IVF treatment. ^b^If maternal income, education or employment was unavailable 1 year before the child was born, the closest available information until baseline was used.

We used the Medical Birth Registry^21^ to identify all children born in Denmark between January 1, 1973, and December 31, 1991 (*n* = 1,142,844), ensuring that all individuals had a possible attained age of at least 30 by the last registry collection date, December 31, 2021 (Figure 1). Data on assisted reproduction (secondary outcome) were available until December 31, 2018, and birth cohorts from 1989 to 1991 were excluded from that analysis. We excluded participants who had died or emigrated before baseline, which was set to the 18th birthday (*n* = 60,861). We compared children with a primary or secondary inpatient, hospital outpatient clinic or emergency department AD diagnosis in the Danish National Patient Registry before baseline (exposed cohort) vs. children without such records (unexposed cohort). The earliest contact date served as the diagnosis date. By design, AD could be diagnosed until December 31, 2009. Because of a lack of clinical data, we defined severe AD as any previous systemic immunomodulatory agent prescription or hospital‐based phototherapy before baseline; otherwise, patients' AD was considered moderate. We defined active disease at baseline as any record indicating active eczema into early adulthood, including hospitalization with a primary or secondary AD diagnosis, one or more systemic agent prescriptions, or two or more topical corticosteroid or calcineurin inhibitor prescriptions within the year before baseline. Because primary care diagnoses were unavailable, the population included primarily patients with relatively severe disease.

### Outcomes

We followed the cohorts until the age of 30 to assess various indicators of socioeconomic position, including earned income, long‐term unemployment, single partnership status and childlessness as pre‐defined primary outcomes (Table 1). The age of 30 was chosen based on data availability, but we also conducted sensitivity analyses to examine outcomes at ages 35 and 40. We also performed annual analyses for ages 18–30. For years with missing data or loss to follow‐up, we used the most recent available information up to that age.

**TABLE 1:** Study outcomes.

Outcome	Description
Labor market outcomes	
Percentile rank of earned income	Total annual net salary at the age of 30, measured as the percentile rank of the income distribution for the entire study population within strata of age, sex and calendar year, to account for age and sex differences and inflation. The main outcome for assessing income level. Analyses by age used the median annual income (converted to Danish 2015 currency values from the World Bank) standardized to income at age 18 years = 100.
Low earned income	Binary variable for whether earned income at the age of 30 ranked in the lower quartile of the income distribution. Secondary outcome for assessing income.
Economic self‐sufficiency	Economic self‐sufficiency in at least one year by the age of 30. Defined as yearly income from all available sources, including social benefits, above a ‘minimum level’ set by Statistics Denmark. Secondary outcome for assessing income.
Long‐term unemployment	Unemployment for at least 50% of a given year at any time before the age of 30. Does not include individuals outside the workforce (students, pensioners or parental leave) or receiving disability pensions. Main outcome for assessing labor market attachment.
Non‐health‐related unemployment^a^	Any non‐health‐related episode of unemployment by the age of 30.
Health‐related work absenteeism^a^	Receipt of any social benefits for health‐related work absenteeism (early retirement, sickness benefit/job clarification, vocational rehabilitee programs, etc.) by the age of 30. Does not include educational benefits and maternity leave.
Sickness benefits^a^	Receipt of sickness benefits by the age of 30. Excludes sickness benefits during pregnancy‐related sickness. Short‐term sick leave spells are not captured.
Disability pension^a^	Disability pension by the age of 30. Granted for people whose work capacity is considered permanently impaired, such that work under ordinary terms or flexible employment is not possible.
Relationship outcomes	
Single partnership status	Never married/cohabitating by the age of 30.
Childlessness	No biological/legal child by the age of 30.
Assisted reproduction	Ever had assisted reproductive technology treatment (e.g. in vitro fertilization treatment) by the age of 30. Secondary outcome for assessing childlessness.

^a^ Analyses were restricted to individuals with a baseline after July 1991, when these outcome data became available.

### Covariables

Our rationale for selecting covariables is described in the conceptual framework in Appendix S1. We included age at AD diagnosis (0–4 or ≥5 years); sex; calendar year at AD diagnosis; attention deficit (hyperactivity) disorder, depression or anxiety; epilepsy; asthma; rhinitis; hand/contact dermatitis; any non‐psychiatric comorbidity (measured with the Charlson Comorbidity Index score), preterm birth, low birth weight, intrauterine/birth asphyxia, chromosomal abnormalities and birth order. We measured childhood socioeconomic position as the maternal income rank (below 25th percentile, 25th–75th percentile or above 75th percentile), education level (lower secondary, upper secondary or higher education) and employment status (unemployed, outside workforce or employed), according to the earliest available information from the year before the child was born to baseline, excluding those with missing data (*n* = 220,346). However, we remained concerned about residual and unmeasured confounding by family‐related factors, including socioeconomic background status, family structure, genetic and early environmental factors, neuropsychiatric traits and health‐seeking behaviours, which might be linked to increased prevalence and severity of AD as well as socioeconomic outcomes.^22^, ^23^ Because siblings usually share many of these characteristics, we conducted a secondary sibling analysis that restricted the comparison to all exposure‐discordant full‐siblings in a family, thereby eliminating confounding from measured and unmeasured factors shared by siblings.

### Statistical analyses

We compared baseline characteristics by exposure status. We used generalized linear models to estimate prevalence ratios as measures of relative risks (RRs) for binary variables (using a Poisson distribution with a log link) and the difference in mean percentile rank of earned income (with the identity link function for linear regression). Because of potential *heteroscedasticity* and non‐*normal* distribution of errors, we used the percentile bootstrap method based on 1000 samples with symmetric distribution to compute 95% confidence intervals (CIs) from linear regression analyses. We fitted regression models of increasing complexity^1^: an unadjusted model (conditioned on family in the sibling comparison),^2^ a minimally adjusted model adding sex and calendar year at baseline,^3^ a comorbidity‐adjusted model adding epilepsy, asthma, rhinitis, any non‐psychiatric comorbidity and birth order (in sibling analyses) and^4^ a fully adjusted model adding maternal income and education level. Maternal employment status was not included because of a strong correlation with income. We stratified the results for main outcomes by age at AD diagnosis, sex, AD severity and activity (for individuals with a baseline after the year 2000, because of treatment data availability), presence of hand/contact dermatitis and maternal income and educational level. We also plotted the median real income (standardized at age 18) and the prevalence of long‐term unemployment, single partnership status and childlessness by age during follow‐up. Finally, we examined the robustness of the results through various sensitivity analyses described in Appendix S3. All analyses were conducted in SAS v9.4.

## RESULTS

### Characteristics

The main analysis included 8409 children with and 853,228 children without hospital‐diagnosed AD before 18 years of age after excluding children who emigrated or died before baseline or who had missing data (study flowchart in Figure S1). Among children with AD, 56.6% were diagnosed before 5 years of age, 46.4% were female, 2.2% met our definition of severe AD, and 20% had active disease at baseline (Table 2). As expected, asthma, rhinitis and hand/contact dermatitis were more common in individuals with rather than without childhood AD, as were other somatic comorbidities and the selected neuropsychiatric disorders. Maternal socioeconomic position was similar between groups. No difference was observed in loss to follow‐up by 30 years of age (2.4% vs. 2.2%). The secondary analysis included 5120 siblings with and 6350 without AD with a more equal distribution of characteristics (Table 2).

**TABLE 2:** Characteristics of children with or without hospital‐diagnosed atopic dermatitis (AD) before 18 years of age, Danish birth cohorts 1973–1991^a^.

	Main analysis		Sibling analysis	
	With AD, no. (%)	Without AD, no. (%)	With AD, no. (%)	Without AD, no. (%)
Total	8409 (100.0)	853,228 (100.0)	5119 (100)	6352 (100)
Age at AD diagnosis^b^				
< 5 years	4758 (56.6)	NA	2841 (55.5)	NA
5–17 years	3651 (43.4)	NA	2278 (44.5)	NA
Sex				
Female	3899 (46.4)	413,363 (48.4)	2368 (46.3)	3114 (49.0)
Male	4510 (53.6)	439,865 (51.6)	2751 (53.7)	3238 (51.0)
Birth year				
1973–1977	1305 (15.5)	217,788 (25.5)	710 (13.9)	1089 (17.1)
1978–1982	1675 (19.9)	205,075 (24.0)	1214 (23.7)	1667 (26.2)
1983–1987	2594 (30.8)	216,140 (25.3)	1831 (35.8)	2027 (31.9)
1988–1991	2835 (33.7)	214,225 (25.1)	1364 (26.6)	1569 (24.7)
Calendar year of AD diagnosis				
1977–1981	1270 (15.1)	NA	730 (14.3)	NA
1982–1986	1576 (18.7)	NA	1087 (21.2)	NA
1987–1991	2016 (24.0)	NA	1271 (24.8)	NA
1992–1996	2034 (24.2)	NA	1155 (22.6)	NA
1997–2001	996 (11.8)	NA	595 (11.6)	NA
2002–2006	444 (5.3)	NA	245 (4.8)	NA
2007–2009	73 (0.9)	NA	36 (0.7)	NA
Setting of AD diagnosis				
Inpatient	5614 (66.8)	NA	3501 (68.4)	NA
Outpatient clinic or emergency department	2795 (33.2)	NA	1618 (31.6)	NA
Severity at baseline^b^				
Moderate	5620 (97.8)	NA	3341 (97.6)	NA
Severe	127 (2.2)	NA	81 (2.3)	NA
Activity at baseline^b^				
Non‐active	4597 (80.0)	NA	2693 (78.7)	NA
Active	1150 (20.0)	NA	729 (21.3)	NA
Epilepsy	222 (2.6)	11,413 (1.3)	114 (2.2)	96 (1.5)
Asthma	4054 (48.2)	75,025 (8.8)	2461 (48.1)	994 (15.6)
Rhinitis	2675 (31.8)	60,239 (7.1)	1625 (31.7)	748 (11.8)
Hand or contact dermatitis at baseline	270 (3.2)	1354 (0.2)	169 (3.3)	31 (0.5)
Non‐psychiatric comorbidity^c^	447 (5.3)	17,041 (2.0)	268 (5.2)	184 (2.9)
Mother's income level				
Low (<Q1)	1365 (16.2)	142,995 (16.8)	781 (15.3)	1093 (17.2)
Moderate (Q1–Q3)	4927 (58.6)	510,391 (59.8)	3151 (61.6)	3794 (59.7)
High (>Q3)	2117 (25.2)	199,842 (23.4)	1187 (23.2)	1465 (23.1)
Mother's highest education level				
Lower secondary education	2544 (30.3)	224,655 (26.3)	1441 (28.2)	1823 (28.7)
Upper secondary education	3661 (43.5)	401,866 (47.1)	2164 (42.3)	2646 (41.7)
Higher education	2204 (26.2)	226,707 (26.6)	1514 (29.6)	1883 (29.6)
Mother's employment status				
Unemployed/disability pension	1088 (12.9)	96,032 (11.3)	632 (12.3)	790 (12.4)
Out of the workforce, including students	1012 (12.0)	96,700 (11.3)	543 (10.6)	771 (12.1)
Employed	6309 (75.0)	660,496 (77.4)	3944 (77.0)	4791 (75.4)
Birth order				
Missing	0 (0)	7 (0.0)		
Not firstborn	4276 (50.9)	428,198 (50.2)	3245 (63.4)	3535 (55.7)
Firstborn	4133 (49.1)	425,023 (49.8)	1874 (36.6)	2817 (44.3)

^a^ See Figure 1 for detailed definitions of cohorts and covariate assessment. Distribution of variables for sensitivity analyses is shown in Table S6.

^b^ Estimated among individuals with baselines in the year 2000 or later (*n* = 5747 exposed in total cohort; *n* = 3422 in sibling cohort) because of treatment data availability.

^c^ According to the Charlson Comorbidity Index.

### Socioeconomic outcomes

**FIGURE 2:**
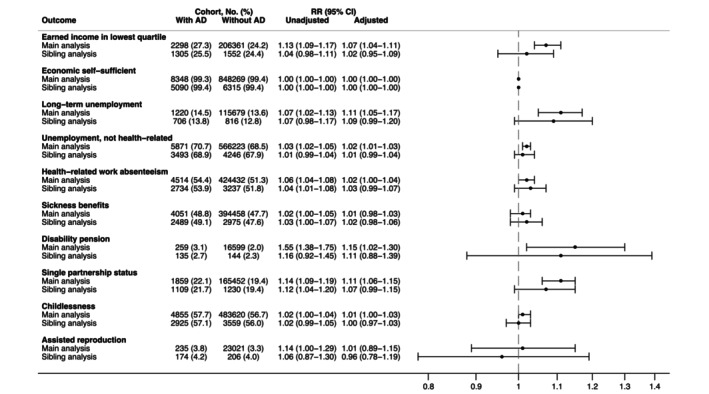
Socioeconomic outcomes by 30 years of age in children with atopic dermatitis compared with children without atopic dermatitis (main analysis) or siblings without atopic dermatitis (secondary analysis). AD, atopic dermatitis; CI, confidence interval; RR, relative risk. The circles with error bars indicate the RR with 95% CIs, adjusted for sex, calendar year at baseline, epilepsy, asthma, rhinitis, any non‐psychiatric comorbidity, mother's income and education level and birth order (in sibling analyses). The sibling comparison was conditioned on the family.

The total population and sibling analyses are summarized in Figure 2 (further details in Tables S2 and S3). Although childhood AD was associated with lower earned income in the total population analysis, the difference was small, with a mean percentile rank of 48.8 (SD 29.5) vs. 51.0 (SD 28.9) in individuals with vs. without childhood AD (fully adjusted mean percentile difference − 1.2; 95% CI: −1.8 to −0.6). Similarly, individuals with childhood AD were slightly likelier to belong to the lowest income quartile (27.3% vs. 24.2%; adjusted RR 1.07; 95% CI: 1.04–1.11). Almost all participants were economically self‐sufficient.

Childhood AD was associated with an RR of long‐term unemployment of 1.11 (95% CI: 1.05–1.17), but the prevalence was less than 1% higher than that in individuals without childhood AD. Moreover, no substantial associations of childhood AD with non‐health‐related unemployment of any duration (70.7% vs. 68.5%; RR 1.02; 1.01–1.03) or health‐related work absenteeism (54.4% vs. 51.3%; adjusted RR 1.02; 1.00–1.04) were observed.

AD was not associated with a higher prevalence of receiving sickness benefits (48.8% vs. 47.7%; RR 1.01; 0.98–1.03). Disability pension was granted to 3.1% of individuals with vs. 2.0% of individuals without AD (unadjusted RR: 1.55; 95% CI: 1.38–1.75). This estimate was substantially attenuated to 1.15 (95% CI: 1.02–1.30) in the fully adjusted model (Figure 2), explained by adjustment for comorbidities (Table S3).

By age 30, 22.1% of patients with AD had never been in a partnership, compared with 19.4% of individuals without AD (RR 1.11; 95% CI: 1.06–1.15). However, no difference was observed in the prevalence of childlessness (57.7% vs. 56.7%; RR 1.01; 95% CI: 1.00–1.03) or assisted reproduction (3.8% vs. 3.3%; RR 1.01; 95% CI: 0.89–1.15).

**FIGURE 3:**
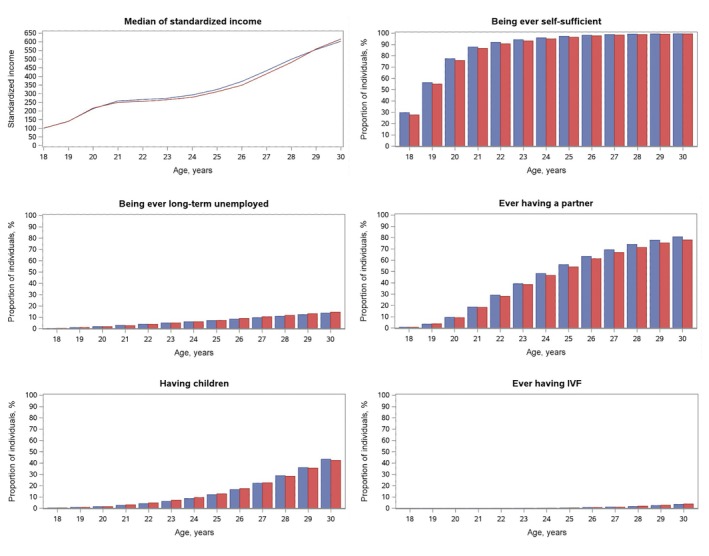
Socioeconomic outcomes by age among children with (red) and without (blue) an atopic dermatitis diagnosis before 18 years of age. Individuals with missing or zero income at age 18 (15% exposed; 17% unexposed) were excluded from the analysis of the median of standardized income.

In the sibling analysis, the absolute and relative measures of differences generally approached the null for all outcomes (Figure 2). Findings were consistent in analyses by age during follow‐up (Figure 3).

### Additional analyses

We observed several subgroup differences for the main outcomes (Tables S4 and S5). Patients with AD and concomitant hand/contact dermatitis had a more pronounced decrease in the mean percentile of earned income (46.1) than those with AD only (48.9) or hand/contact dermatitis only (51.0) compared with those without atopic, hand or contact dermatitis (51.0). The association between AD and partnership was explained primarily by an elevated risk of being single in men (28.3% vs. 24.3%; RR 1.14; 95% CI: 1.09–1.19) and individuals with severe eczema (26.0% vs. 19.8%; RR 1.35; 95% CI: 1.01–1.81), hand/contact dermatitis (27.8% vs. 19.4%; RR 1.41; 95% CI: 1.17–1.71) or a mother with lower secondary education (22.6% vs. 18.0%; RR 1.18; 95% CI: 1.09–1.26). Patients with severe AD had a higher risk of childlessness than individuals without AD (63.0% vs. 57.2%; RR 1.14; 95% CI: 1.00–1.30).

Sensitivity analyses confirmed the main findings (Tables S6–S10) with some noticeable exceptions. Estimates generally attenuated toward the null in analyses excluding patients who did not contribute to the sibling analysis. Furthermore, the association between AD and an increased risk of childlessness emerged at older ages. There was a 2.5% absolute difference in the prevalence by age 35 (RR 1.05; 95% CI: 1.01–1.10) as well as by age 40 (RR 1.06; 95% CI: 0.99–1.13). For assisted reproduction, the RR was 0.94 (95% CI: 0.84–1.06) by age 35 and 1.01 (95% CI: 0.88–1.17) by age 40, but the absolute differences reached only 0.2%.

## DISCUSSION

Using nationwide population‐based registries and triangulation of data from population and sibling designs, we found no substantial evidence that Danish children with hospital‐diagnosed AD have poorer labor market outcomes overall; however, these individuals had a slightly elevated likelihood of receiving disability pensions, at least partly because of comorbidities. The probabilities of partnership or parenthood were diminished in certain AD subgroups, including those with severe eczema. However, our sibling analysis suggested that family‐related confounding may have explained the small differences observed in the total population analysis.

Cross‐sectional studies have indicated diminished productivity in patients with AD, owing to absenteeism, and particularly presentism, at work or school.^2^ However, we observed no association between moderate‐to‐severe childhood AD and labor market outcomes, thus supporting most,^13^, ^14^, ^15^, ^16^ but not all,^17^ survey‐based cross‐sectional studies on income. Cross‐sectional studies examining associations between AD and employment status are more conflicting.^14^, ^15^, ^17^ A possible explanation for the absence of association between AD and long‐term labor market outcomes is that patients might continue working during flares or receive treatments or guidance (e.g. to avoid careers with a high risk of hand eczema), which could mitigate the negative effects of AD on labor market attachment. The lower earned income observed in individuals with concomitant hand or contact dermatitis highlights the value of such counselling. Nonetheless, evidence remains mixed regarding whether AD affects educational and occupational choices, and most studies have found no association with job changes.^18^

Matched cohort studies outside Denmark have indicated that individuals with rather than without AD have a higher prevalence of any sick leave (70% vs. 67%)^8^ or sick leave for >7 days because of unspecified eczema (9.7% vs. 1.9%),^9^ and higher mean indirect work‐loss costs per month because of employer disability payments and sick leave ($148 vs. $85).^10^ One study has suggested hand eczema as a possible explanation.^9^ Unfortunately, the studies' validity might have been affected by loss to follow‐up,^9^ recruiting methods for patients with AD (e.g. only those undergoing prick‐testing)^8^, ^9^ or comparators (e.g. patients with other skin diseases),^8^, ^10^ and confounding.^8^, ^9^, ^10^ The different outcomes also hinder comparison with our study. A Danish cohort study in 28,156 people with and 473,836 people without hospital‐diagnosed AD associated severe AD with an adjusted hazard ratio of 1.07 (1.02–1.13) for any social benefits and 1.67 (1.45–1.93) for disability pension in people born in 1964–1976 (most complete follow‐up).^11^ The odds ratio for any sickness benefit was 1.32 (95% CI: 1.13–1.54), and benefits lasted 3 weeks on average in patients with severe AD vs. 2 weeks in comparators. Despite using the same data sources as our study, methodological shortcomings limit interpretability and might explain the stronger associations observed. Future information was used to ascertain exposure (including severity) and sample comparators, thus possibly introducing selection and immortal‐time bias. The youngest birth cohorts could not contribute any outcomes because they had not reached adulthood during their study. Finally, residual and unmeasured confounding by childhood socioeconomic position and comorbidities might have occurred. Our results suggested that patients' increased risk of disability pensions was due to comorbidity rather than AD alone, although both studies lacked specific data regarding reasons for disability pensions. Notably, pensions due to AD in Denmark are low overall but increased from an average of 4.2 per year in 1970–1976 to 18.0 per year in 1970–2002 and 14.2 per year during 2003–2008.^24^, ^25^

Findings regarding AD and long‐term relationship outcomes are conflicting. A sibling‐matched study from Sweden and Finland has found no association between hospital‐diagnosed AD and having a partner by age 50 in men (OR 1.01 [0.88–1.16]) and age 45 in women (OR 1.02 [0.90–1.15]).^12^ No association between AD and not having biological children (OR 1.05 [0.91–1.21] in men; 0.95 [0.83–1.09] in women) was observed. A population‐based U.K. primary care study has found slightly higher fertility rates in women with vs. without a primary care AD diagnosis (59.4 vs. 51.0 live‐births per 1000 person‐years; adjusted ratio: 1.15; 95% CI 1.13–1.17).^26^ Our study's absolute measures and subgroup analyses suggested clinically relevant differences in the prevalence of 4–8% for partnership and childlessness in, for example men and individuals with severe childhood AD. Stigmatization and concerns regarding heritability, the effects of systemic treatments and the challenges in managing AD during pregnancy and parenthood might have roles. Economic uncertainty is less likely, given the limited association with earned income. Rather than indicating stigmatization, our findings might reflect personal priorities regarding work–life balance, for example pursuing higher education before union and family formation.^27^ However, post hoc analyses (Table S11) show that educational status was lower in those who were single or childless until age 40, especially for those who had childhood AD. These analyses further indicated that single partnership status mediates the risk of childlessness. No clear biological evidence links AD to fertility issues, and we observed no association with assisted reproduction.

Key strengths of our study include the use of a nationwide, population‐based cohort, the inclusion of both general population and sibling comparators, and access to detailed long‐term socioeconomic data extending into early adulthood for birth cohorts spanning three decades. However, several limitations remain, including potential bias toward the null due to misclassification of exposure (from including mild AD among unexposed individuals) and outcomes. We included earned income to reflect patients' ability to earn a living and maintain employment, which does not account for the financial burden of decreased disposable income because of treatment costs. We might have missed an association between AD and shorter sick leave periods because we measured absenteeism qualifying for social benefits. Because our definition might have classified some roommates as romantic partners, the association with partnership might have been partly mediated by any stigmatization affecting housing and close friendships.

We addressed family‐related confounding through sibling analyses, which provided reassuring results for children with AD. Although this approach has limitations, for example potentially amplifying confounding by less shared factors (than AD),^22^ we attempted to adjust for such factors (e.g. birth order and calendar year) in our analyses and by restricting the sibling age gap. Although the effect of AD on well siblings is unclear,^28^, ^29^ the results might have been attenuated if siblings' socioeconomic outcomes were negatively affected.^23^ Bias from other ‘sibling carryover’ seems unlikely because of the small age gap and ascertainment of outcomes in adulthood. Exposure misclassification can be more pronounced than that in population analysis,^22^ but excluding patients receiving topical AD treatment had a limited impact on results. The sibling analyses also had lower precision than the main analyses. Finally, the sibling results may not be generalized to all family structures, as our sensitivity analyses suggested a possible stronger negative impact of AD in families without (full) siblings.

We would expect weaker associations in cohorts with milder AD. However, our therapy‐based severity definition might not have captured variations in stigma associated with, for example itching, visibility of lesions and genital involvement,^3^, ^6^, ^30^ leading to an underestimate of the impact of severe disease. Denmark's healthcare system, social safety net and patient education programs (e.g. eczema schools) might have mitigated AD's negative effects, reducing generalizability to settings with less access to such support or other cultural relationship norms. Moreover, newer systemic therapies (e.g. dupilumab) that were unavailable during the study period have shown positive effects on, for example work productivity and sexual health.^31^, ^32^, ^33^

In conclusion, our findings are reassuring, showing that moderate‐to‐severe childhood AD is not associated with a reduced ability to earn a living and maintain long‐term employment in adulthood within a universal welfare system. However, individuals with AD had an elevated likelihood of receiving disability pensions, likely due to comorbidities, and a reduced likelihood of partnership and parenthood. These findings highlight the importance of a holistic approach to AD management, which should consider patients' broader life situations, helping to support them as they navigate adulthood and establish relationships or families.

## AUTHOR CONTRIBUTIONS

**Sigrun A. J. Schmidt:** Conceptualization‐Lead, Funding acquisition‐Lead, Investigation‐Lead, Methodology‐Lead, Project administration‐Lead, Writing – original draft‐Lead. **Dóra Farkas:** Conceptualization‐Supporting, Data curation‐Lead, Formal analysis‐Lead, Investigation‐Supporting, Methodology‐Lead, Supervision‐Equal, Writing – review & editing‐Equal. **Mette Deleuran:** Conceptualization‐Supporting, Funding acquisition‐Supporting, Investigation‐Supporting, Methodology‐Supporting, Supervision‐Equal, Writing – review & editing‐Equal. **Christian Vestergaard:** Conceptualization‐Supporting, Funding acquisition‐Supporting, Investigation‐Supporting, Methodology‐Supporting, Supervision‐Equal, Writing – review & editing‐Equal. **Niels Skipper:** Conceptualization‐Supporting, Investigation‐Supporting, Methodology‐Supporting, Supervision‐Equal, Writing – review & editing‐Equal. **Henrik Toft Sørensen:** Conceptualization‐Supporting, Funding acquisition‐Supporting, Investigation‐Supporting, Methodology‐Supporting, Resources‐Lead, Supervision‐Equal, Writing – review & editing‐Equal. **Sinead Langan:** Conceptualization‐Supporting, Funding acquisition‐Lead, Investigation‐Supporting, Methodology‐Supporting, Supervision‐Equal, Writing – review & editing‐Equal.

## FUNDING INFORMATION

SML received funding from a Wellcome Trust Senior Research Fellowship in Clinical Science (205039/Z/16/Z). The funders had no role in study design, data collection and analysis, decision to publish or preparation of the manuscript. The contents are solely the responsibility of the authors and do not necessarily represent the official views of the funders. For the purpose of Open Access, the author has applied a CC BY public copyright licence to any Author Accepted Manuscript (AAM) version arising from this submission.

## CONFLICT OF INTEREST STATEMENT

The Department of Clinical Epidemiology, Aarhus University, receives funding for other studies in the form of institutional research grants to (and administered by) Aarhus University. The Department of Clinical Epidemiology, Aarhus University, confirms that none of these studies has any relation to the present study. No other disclosures are reported.

## ETHICAL APPROVAL

The study was approved by the Danish Data Protection Agency (record number 2016–051‐000001; serial number 605).

## ETHICS STATEMENT

Danish legislation does not require approval from an ethical review board or informed consent from participants in registry‐based studies. Small sample sizes (<5) were omitted to obscure the values and preserve anonymity. Results are reported per STROBE and RECORD guidelines.

## Supporting information


Data S1.

## Data Availability

No additional unpublished data are available, because this study used existing data from the Danish nationwide registries that are accessible only to researchers meeting local requirements, after approval by the Danish Data Protection Agency, the Danish Health Data Authority and Statistics Denmark.
