# Supplementary material for: Long‐term socioeconomic outcomes in Danish children with moderate‐to‐severe atopic dermatitis
Source: J Eur Acad Dermatol Venereol. 2025 Oct 3;40(1):79–89. doi: 10.1111/jdv.70085 (PMC12723571; doi:10.1111/jdv.70085)
Supplement: Supplementary file 1 — Data S1. [file JDV-40-79-s001.docx]

**Supplementary Material**

**Long-term socioeconomic outcomes in Danish children with moderate-to-severe atopic dermatitis**

Sigrun Alba Johannesdottir Schmidt, PhD;^1,2^ Dóra Körmendiné Farkas, MSc;^1^ Mette S. Deleuran, DMSc;^2^ Christian Vestergaard, DMSc;^2^ Henrik T. Sørensen, DMSc;^1^ Niels Skipper, PhD;^3^ Sinéad M. Langan, PhD^4,5^

*^1^ Department of Clinical Epidemiology and Center for Population Medicine, Aarhus University Hospital and Aarhus University, Aarhus, Denmark*

*^2^ Department of Dermatology, Aarhus University Hospital, Aarhus, Denmark*

*^3^ Department of Economics and Business Economics, Aarhus University, Aarhus, Denmark*

*^4^ Faculty of Epidemiology and Population Health, London School of Hygiene and Tropical Medicine, London, United Kingdom*

*^5^ Health Data Research UK*

#

Contents

[Appendix 1. Conceptual framework 3](#_Toc202868943)

[Appendix 2. Data sources 4](#_Toc202868944)

[Appendix 3. Description of sensitivity analyses 5](#_Toc202868945)

[Table S1. Definition of study variables 6](#_Toc202868946)

[Table S2. Comparison of the percentile of the income distribution at age 30 years in children with atopic dermatitis compared with children without atopic dermatitis (main analysis) and siblings without atopic dermatitis (secondary analysis) 11](#_Toc202868947)

[Table S3. Number of events, persons at risk, prevalence, and relative risks with 95% confidence intervals (CIs) of various socioeconomic outcomes by age 30 in children with atopic dermatitis compared with children without atopic dermatitis (main analysis) and siblings without atopic dermatitis (secondary analysis) 12](#_Toc202868948)

[Table S4. Association between atopic dermatitis (AD) and percentile of the income distribution at age 30 according to subgroups of age at AD diagnosis, sex, AD severity, AD activity, hand or contact dermatitis, and maternal socioeconomic position 13](#_Toc202868949)

[Table S5. Association between atopic dermatitis (AD) and long-term unemployment, single partnership status and childlessness by age 30 according to subgroups of age at AD diagnosis, sex, AD severity, AD activity, hand or contact dermatitis, and mother’s socioeconomic position 14](#_Toc202868950)

[Table S6. Distribution of variables used for sensitivity analyses 16](#_Toc202868951)

[Table S7. Sensitivity analyses for the association between atopic dermatitis (AD) and percentile of the income distribution at the age of 30. 17](#_Toc202868952)

[Table S8. Sensitivity analyses for the association between atopic dermatitis and various socioeconomic outcomes by age 30. 18](#_Toc202868953)

[Table S9. Sensitivity analyses for the association between atopic dermatitis (AD) and percentile of the income distribution at age 30, sibling comparison 22](#_Toc202868954)

[Table S10. Sensitivity analyses for the association between atopic dermatitis and various socioeconomic outcomes by age 30, sibling comparison 23](#_Toc202868955)

[Table S11. *Post hoc* analyses of the distribution of education level and partnership status by the outcomes of partnership and childlessness in those with and without childhood atopic dermatitis (AD) 24](#_Toc202868956)

[Figure S1. Flowchart 25](#_Toc202868957)

[References 26](#_Toc202868958)

# **Appendix 1. Conceptual framework**

We hypothesized that atopic dermatitis (AD) is associated with negative labor market outcomes (income, economic self-sufficiency, employment, work absenteeism, sickness benefits, and disability pension) and relationship outcomes (singlehood, childlessness, and assisted reproduction).

A directed acyclic graph (DAG) for the hypothesized relationships is shown below. For simplicity, the various socioeconomic outcomes have been combined in the figure. There is also a possible interplay between the study outcomes, which are not depicted. Thus, the potential effect of AD on the income outcome is influenced by employment status and could also depend on partner status, as single people may require a higher income to cover material purchases independently and may have different capacity and incentive to devoted time to education. Employment as an outcome may similarly be influenced by educational attainment, as people with lower education levels are more likely to be unemployed. The social outcomes (partnership, childlessness, and assisted reproduction) may also be influenced by educational levels, as individuals pursuing longer education often postpone cohabitation, marriage and procreation. Income may also affect these outcomes, e.g. if people decide to cohabitate or marry for economic reasons. Partner status directly influences the likelihood of the outcomes of childlessness and assisted reproduction.


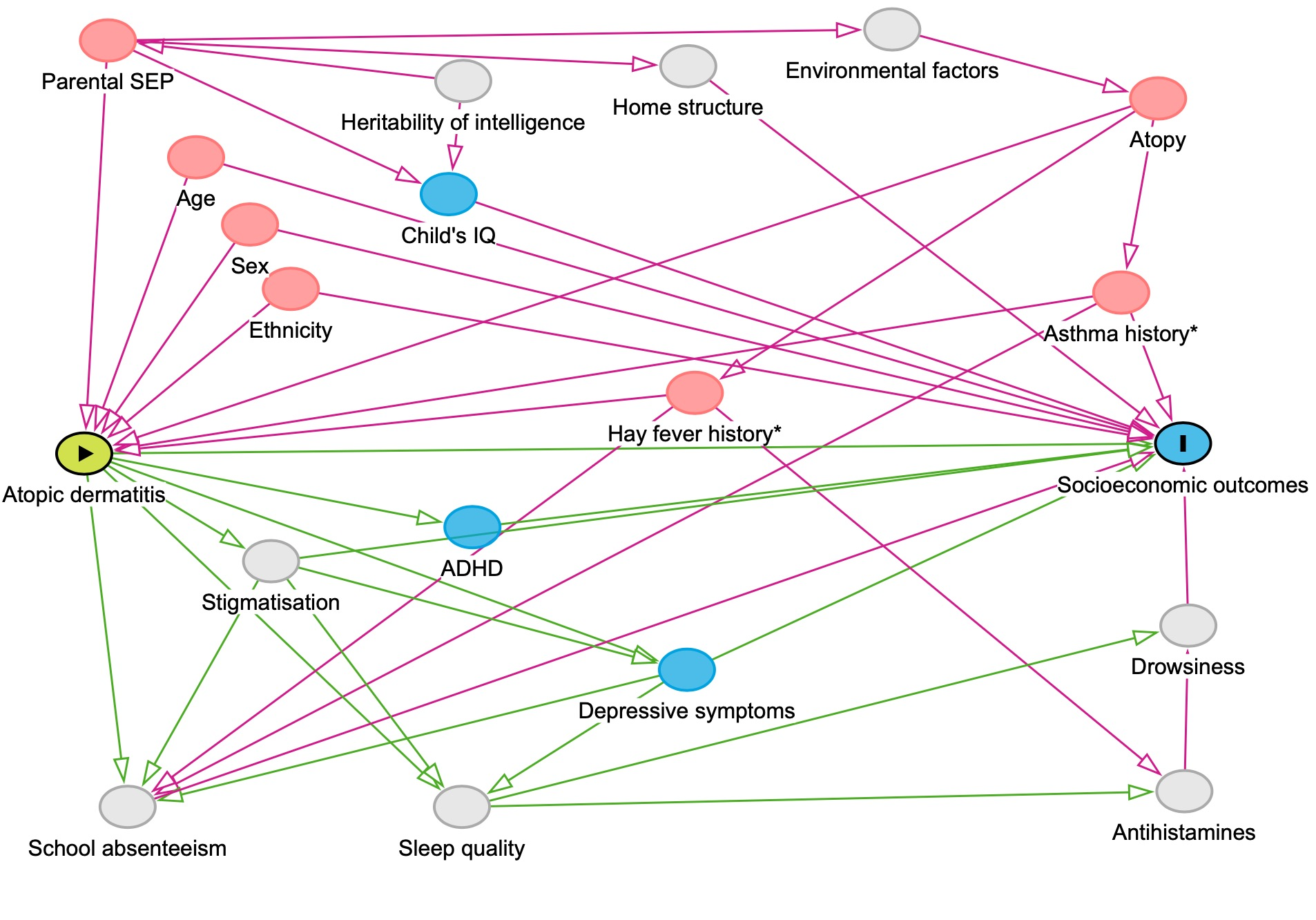


#

Abbreviation: ADHD = attention deficit hyperactivity disorder; SEP = socioeconomic position

# **Appendix 2. Data sources**

We based the study on data from several nationwide health and social registries, described below. We linked the registries at the individual level using the unique personal civil registration number assigned to all Danish residents at birth or immigration.^1^ Statistics Denmark, the central authority on Danish statistics, collected and linked data for the current study from the different registries. SAJS and DKF received access to the de-identified data through Statistics Denmark’s secure servers, where DKF performed sampling of cohorts, data cleaning, and analyses.

The Danish Medical Birth Registry has recorded data on all live- and stillbirths in Denmark since Jan 1, 1973.^2^ It includes data on pregnancy, delivery, and outcome.

The Civil Registration System includes all persons residing in Denmark since Jan 1, 1968.^1^ Individuals’ birth date, sex, place of residence, last immigration and emigration dates, and the identity of parents/siblings are recorded. Almost all first degree-relatives are identifiable for people born in 1950 or later.

The Danish National Patient Registry includes data from outpatient and inpatient visits in hospital-based care, including date of visit, primary and secondary diagnoses, procedures, and certain hospital-based treatments (e.g., methotrexate injections).^3^ Inpatient contacts are available since 1977, outpatient visits since 1995, and hospital-based treatments since 1999. The diagnosis is coded according to the ICD-8 system (until 1993) and ICD-10 (thereafter) by the treating physician.

The Danish Psychiatric Central Research Registry includes all admissions to psychiatric wards since 1970.^4^ Data have been merged with the Danish National Patient Registry since 1994.

The Danish National Prescription Registry, established on Jan 1, 1995, records prescriptions dispensed at community pharmacies, including data on the drug, user, prescriber, and pharmacy.^5^

The In Vitro Fertilisation (IVF) registry has registered treatments with assisted reproduction techniques (e.g. IUI and IVF) at public and private fertility clinics since Jan 1, 1994.^6^

The Population Education Registry includes data on the highest completed education, including the time of achievement since December 1, 1980.^7^

The Integrated Database for Labor Market Research (IDA) holds labor market information recorded on both the individual level (e.g., occupation, earning, seniority) and on the establishment level (e.g., line of business, year of establishment).^8^ It includes more than 200 variables. Income data are based on the Salary Information Registry. The registry covers the Danish population on January 1 each year since 1980 (labor market attachment since November 1980).^8^

The employment classification module includes information on occupation and employment status based on the activity with the highest income throughout the year (considered the most important place of employment) since 1976.^8^ Information covers people chargeable with tax in Denmark on January 1 each year. It is based on reports from companies, public payroll systems, and other registries, including the Salary Information Registry and the Central Taxpayer’s Registry.

Danish Register for Evaluation of Marginalization (DREAM) includes information on public transfer payments since July 1991.^9^ It covers all Danish residents who have received social benefits or any other transfer income for at least one day in a given week. People granted disability pensions before 1994 and public retirement pensioners who have not received other transfer income are not included.

# **Appendix 3. Description of sensitivity analyses**

We examined the robustness of the main results through several sensitivity analyses:

1. Repeated analyses to assess outcomes at the ages 35 and 40.
2. Addressed missing outcome data by excluding individuals with any year of missing data.
3. Addressed missing income data using peak income instead of income at age 30.
4. Additionally adjusted for attention deficit disorder, depression, and anxiety, to explore their roles as mediators.
5. Additionally adjusted for a composite score of childhood socioeconomic position, based on maternal income, educational level, and employment status.
6. Repeated the analyses after reclassifying missing maternal education level as “no education” (below lower secondary education) to further address potential residual confounding from childhood socioeconomic position.
7. Excluded individuals born preterm, with low birth weight, intrauterine/birth asphyxia, or chromosomal abnormalities.
8. Excluded stillbirths from the analysis of childlessness.
9. Excluded patients with atopic dermatitis who did not contribute to the sibling analysis to explore whether differences in sibling results were due to exclusion of families with only one child or half-siblings (potentially suggesting effect modification by family structure).
10. Repeated the analysis, adjusting for the highest parental income and educational level (based on whichever parent had the higher value using the earliest available data), instead of using maternal information only. We performed this analysis in the same cohort as the main analysis for comparability. We used maternal data in the main analysis under the assumption that they are more closely correlated to pre- and perinatal exposures and early-life caregiving, and can be more complete.
11. Restricted the cohort to individuals with a baseline date in 1996 or later, ensuring at least one year prescription and outpatient diagnosis history. We then reanalyzed data before and after excluding unexposed individuals without prior prescriptions for topical corticosteroids or calcineurin inhibitors, to assess the impact of possible misclassification of individuals with mild eczema among the unexposed.
12. Repeated the sibling analysis restricted to siblings with ≤3 years’ age difference, to decrease residual confounding from time-related changes in family factors and calendar time.
13. Repeated the sibling analysis adding the requirement that siblings had no prescriptions for topical corticosteroids or calcineurin inhibitors before baseline, to avoid misclassifying siblings with mild atopic dermatitis as unexposed.

#

# **Table S1. Definition of study variables**

| Variable | Coding | Description |
| --- | --- | --- |
| Exposure (atopic dermatitis) | | |
| Hospital diagnosis | ICD-8: 691 or ICD-10: L20 | Inpatient, hospital outpatient clinic and emergency department diagnoses with atopic dermatitis as a primary or secondary diagnosis in the Danish National Patient Registry, including ongoing contacts. Diagnoses from all specialties were considered for completeness. ICD-8 diagnoses recorded with an additional (“modification”) code indicating that it potentially an uncertain/unverified/working diagnosis (c_diagmod=1–7) were excluded. |
| Severe disease | - In the Danish National Patient Registry, procedure codes: “BNGA1” “BNGA2” “BNGA3” “BNGA4” “BOHJ18B8” “BWHB83” “BWHA115” “BOHJ20” “BOHJ22” or - In the Danish National Prescription Registry, ATC codes: “L04AX01” “L01BA01” “L04AX03” “L04AD01” “L04AA06” “D11AH05” | Severe, if any of codes for systemic immunomodulatory treatment (cyclosporine, azathioprine, mycophenolate, methotrexate) or phototherapy.  Moderate otherwise |
| Active disease | 1) a hospitalization (inpatient contact) with atopic dermatitis as a primary or secondary diagnosis  2) at least one prescription for a systemic agent for dermatitis (see severe atopic dermatitis above)  3) a topical corticosteroid or calcineurin inhibitor identified in the Danish National Prescription Registry using ATC codes (see below) | Active if any of the relevant codes within 12 months before baseline.  Not active otherwise |
| Treatment with topical steroid/calcineurin inhibitor | ATC codes: “D07” “D11AH01” “D11AH02” | Any prescription for a topical steroid/calcineurin inhibitor in the Danish National Prescription Registry. For sensitivity analysis of the sibling comparison. |
| Labor market outcomes | | |
| Earned income | Income recorded in variable LONIND (until 2008) and SAMLET_BREDT_LOEN_BELOEB (2008 and onwards) | Total annual net salary in the final year of follow-up (i.e., at age 30 years), based on reports to the Danish tax system. It does not include e.g. transfer income and other social benefits. We computed the rank percentile (values 1–99) of earned income at age 30 using the income distribution for the entire study population within strata of age (30 years in main analysis), sex, and calendar year. This approach accounts for general economic development during the study and differences in age and sex, and it is easier to understand than an absolute difference in Danish crowns or other specific currencies. We included all values (including null values) in the calculation. To ensure consistency in our findings, we included as secondary measures of income:  (1) a binary variable for whether income ranked in the lower income quartile or not;  (2) the change in median annual income from age 18 to 30 years using a standardized index with annual income at age 18 years = 100. For this analysis, we converted earned income to the 2015 currency values for Denmark using the GDP deflator tool downloaded from the World Bank (i.e., real income).  (3) economic self-sufficiency (see below) |
| Economic self-sufficiency | Variable BESKST13=”01” ”02” ”03” ”04” ”05” ”06” ”07’” ”09” ”10” ”11” or ”12”=self-sufficient | Having ever had one full year of economic self-sufficiency by age 30. Definition is based on the Employment Classification Module, Statistics Denmark, which classifies people based on their most important source of income during the year. Persons who are not self-sufficient have a personal yearly income (from all available sources, including business income and various social benefits) that is below a ‘minimum level’ defined by Statistics Denmark. This level corresponds to 50% of a student’s annual minimum subsidy adjusted by the consumer price index (e.g., 49,138 DKK in 2005 and 60,000 DKK in 2017). |
| Long-term unemployment | Variable BESKST13=”10” | Unemployment at least 50% of a given year (“net unemployment”) at any time before age 30. Excludes those outside the work force (students, pensioners, parental leave) and those receiving disability pension. |
| Unemployment, not health-related, any duration | Any weekly entry in the DREAM database^c^ with codes 111–115, 124-126, 130-149, 151-153, 160, 163-169, 211-219, 221-222, 224-225, 231-232, 297-299, 414, 511, 522, 541, 700, 703-739, 741-742, 751-752, 759 (entries before 1 January 1997 recoded as 299->115) | Any episode of unemployment that is not health-related by age 30 years, as identified through the DREAM database, which records weekly updated information on various social benefits. In this registry, a person is recorded as unemployed in each week if unemployed for at least one day. |
| Health-related work absenteeism, any duration | Any weekly entry in the DREAM database^c^ with codes: 622, 740, 743-748, 750, 753-758, 760-768, 769, 771-774, 779, 781-785, 791-793, 796, 797, 810, 813-819, 870, 873-879, 890-899, 895 (entries before 1 December 2010 recoded as: 753->734, 754->706, 755+756->705, 757->704) | Having ever received any social benefit for health-related work absenteeism (e.g., sickness benefit/job clarification and disability pensions) by age 30. Educational benefits and maternity leave were not included. The database only captures absence that qualifies for social benefits such as sickness absence compensation by the state (beginning after 14-30 days of sick leave, depending on calendar year and employment). Shorter sick leave spells are not captured. |
| Sick leave benefit, any duration | Any weekly entry in the DREAM database^c^ with codes for sick leave benefit (entries: 774 890 891-899) | Having ever received sickness benefit by age 30. Sick leave benefits during pregnancy-related sickness were not included. |
| Receipt of disability pension | Any weekly entry in the DREAM database^c^ with codes for disability pension (entries: 622 781 783 797 793) | Having ever received disability pension by age 30. Includes disability pensions (“Førtidspension”) and early retirement pensions after flexible job ("flexydelse”). |
| Relationship outcomes | | |
| Partnership status (single vs. married/cohabitating) | Single: missing data on variables AEGTE_ID, EFALLE and C_faelle_id  Married/cohabitating: non-missing data | Having never been married/cohabitating by age 30. Based on yearly updated information on partnership status as defined by Statistics Denmark according to an algorithm that includes data on civil status, exact address, kinship etc. |
| Childlessness | No childbirth/parenthood registered in the Medical Birth Registry or in the Civil Registration System | Having no children by age 30. Of note, the Civil Registration System includes legal parenthood (e.g., does not differentiate between adoptive and biological children). |
| Assisted reproduction | Cpr number recorded in the IVF registry in variable v_i_mcpr (cpr number for treated) or v_partners_cpr (cpr number or partner, including female partners) | Having ever had assisted reproductive technology treatment (*e.g.*, *in vitro* fertilization treatment) by age 30. This outcome serves as a secondary outcome for assessing childlessness. |
| Covariables | | |
| Age at atopic dermatitis diagnosis | For subgroup analyses: 0–4 years; ≥5 years | Early-onset AD included diagnoses up to age 5 to allow for diagnostic/referral delay until registration in the hospital setting. |
| Sex | Female or male |  |
| Calendar year at baseline | Included as an integer variable in analyses and in 5-year periods for descriptive purposes |  |
| Attention deficit disorder, depression, or anxiety disorder | ICD-8: ”29609” ”29629” ”29699” ”29809” ”30049” ”30009” ”30019” ”30029”;  ICD-10: “F32” ”F33” ”F40” ”F41” "F900” ”F920” ”F931” ”F932” ”F9380”;  ATC: “N06BA” | Identified in the Danish National Patient Registry, the Danish Psychiatric Central Research Registry and the Danish National Prescription Registry. The conditions were combined in one variable due to small numbers. |
| Non-psychiatric comorbidity | Any comorbidity according to the Charlson Comorbidity Index, excluding codes for asthma to avoid overall with asthma variable (below) | Identified in the Danish National Patient Registry |
| Epilepsy | ICD-8: “345” or  ICD-10: “DG40” “DG41” in the Patient Registry | Identified in the Danish National Patient Registry |
| Asthma | ICD-8: “493” in the Danish National Patient Registry  ICD-10: “DJ45” or “DJ46” in the Patient Registry  ATC: “R03” | Identified in the Danish National Patient Registry or the Danish National Prescription Registry as at least one diagnostic code or two prescription fills for drugs against obstructive lung disease |
| Rhino-conjunctivitis | ICD-8: “507”;  ICD-10: “DJ30” “DH101” “DH104A”  ATC: “R01AC” “R01AD” or “S01GX” | Identified in the Danish National Patient Registry or the Danish National Prescription Registry as at least one diagnostic code or two prescription fills for nasal antihistamines/corticosteroids or anti-llergic eye drops |
| Hand/contact dermatitis | ICD-8: ”692”  ICD-10: “DL308H” “DL208D” “DL23” “DL24” ”DL25” | Identified in the Danish National Patient Registry |
| Preterm birth (<37 gestational wk) | v_svlangde (table ’t_lfoed’),  Gestationsalder_dage (table ’MFR’), or  Gestationsalder_uger (table ‘Hjemmefoedsler_blanket’) | Yes, if v_svlangde, Gestationsalder_uger, or Gestationsalder_dage/7 is <37  No otherwise |
| Low birth weight (<2500 g) | V_vagt (table ’t_lfoed’),  vaegt_barn (table ’MFR’), or  vaegt_barn (table ‘Hjemmefoedsler_blanket’) in Medical Birth Registry | Yes, if any of variables with value <2500 g  No otherwise |
| 5-min Apgar score <7 or intrauterine/birth asphyxia | V_apgar5  (table ‘t_lfoed’),  Apgarscore_efter5minutter (table ’MFR’), or Apgarscore_efter5minutter (table ‘Hjemmefoedsler_blanket’) in Birth Registry; ICD-8 code “776” or ICD‐10  code “DP20” or “DP21” in Patient Registry | Yes, if V_apgar5 or Apgarscore_efter5minutter <7 or any of the ICD-8/10 codes.  No otherwise |
| Chromosomal abnormalities (as defined by Eurocat standard code list at the department) | ICD-8 “7593” “7594” “7595”; ICD-10: “DQ9”  in the Patient Registry | Yes, if any of the relevant codes.  No otherwise |
| Birth order | First born; not first born; unknown | 1. First born  2. Not first born  3. Unknown |
| Maternal socioeconomic position^d^ |  |  |
| - Income rank | Percentile rank of annual real income (variable LONIND deflated to 2015) according to the 2015 income distribution for women of the same age. | Categorized as:  1. Low = Income below lower quartile (income<25^th^ percentile)  2. Medium = Income from lower to upper quartile (income=25^th^-75^th^ percentile)  3. High = Income above the upper quartile (income>75^th^ percentile) |
| - Education level | - Lower secondary education: **AFSP1H** or **HOVEDOMRAADE_OVER** = 10 or 29 AND **HFAUDD** 210, 1009, 1010, 1011, 1109, 1110, 1111 or 1209 - Upper secondary education: **AFSP1H** or **HOVEDOMRAADE_OVER** = 20, 25, 30, 35, or 39 - Higher (tertiary) education: **AFSP1H** = 40, 50, 60, 65, or 70 or **HOVEDOMRAADE_OVER** = 40, 50, 60, 70, or 80 | Categorized as:  1. Low = Lower secondary education (Corresponds to ISCED-2011 level 2);  2. Medium = Upper secondary education (Corresponds to ISCED-2011 level 3);  3. High = Higher (tertiary) education (Corresponds to ISCED-2011 levels 5–8);  Note: Those with no education recorded were considered missing. It is not possible with certainty to differentiate between those who have truly missing data on education and those who have no education (did not finish basic schooling). In a sensitivity analysis, we kept those with missing data on maternal education and added a 0 category for “No education” assuming that missing information corresponded to not completing lower secondary school. |
| - Employment status | - Unemployed: variable PSTILL = 40 or 93 or PSOC_STATUS_KODE = 200 or 411 - Outside the workforce: variable PSTILL = 41–43, 45–52, 55–57, 90–92, or 94–98 or PSOC_STATUS_KODE = 300–322, 412–517 or 361 - Employed: variable PSTILL = 01–05, 11–14, 19–20, 31–37, or 71–77 or PSOC_STATUS_KODE = 110–138, 351, 611, 612 | Categorized as:  1. Low = unemployed  2. Medium = outside the workforce, including persons in an educational program, those in early retirement and those receiving other types of public support  3. High = employed |
| - Composite score for maternal socioeconomic position | A composite score of the variables for the mother’s income, education and employment status, as defined above. | Individuals were assigned a score of 1–3 for their category of income, education and employment status. These scores were then combined to a composite score ranging from 3 to 9, categorized as low (scores of 3 and 4) medium (scores of 5 to 7) or high socioeconomic position (scores of 8 and 9). |
| - Paretal income rank and education level | Coded as for maternal income and education above, but using the earliest non-missing data available for either the mother or father, whichever highest. | Used for sensitivity analysis. |
| Exposure-discordant siblings | Persons with the same mother and father but discordant exposure status. A stratum of exposure-discordant siblings could include more than two persons if other eligibility criteria were fulfilled. | Kinship was ascertained in the Civil Registration System |

Abbreviations: ATC: Anatomical Therapeutic Chemical code; ICD-8: International Classification of Diseases version 8; ICD-10: International Classification of Diseases version 10.

All subcodes were included unless otherwise stated; all types of contacts (inpatient, outpatient and emergency) and both primary and secondary diagnoses were considered, regardless of specialty. We used admission/prescription/record date for all variables.

^a^Excluding AFSP1H/DISCED15 “10” codes, which do not lead to a qualification: “1” “200” “205” “1006“ “1007“ “1008“ “1021“ “1022“ “1023“ “1100“ “1101“ “1102“ “1103“ “1104“ “1105“ “1106“ “1107“ “1108“ “1120“ “1121“ “1122“ “1123“ “1206“ “1207“ “1208“ “1410“ “1423“ “1509“ “1510“ “1522“ “1523“ “1721“ “1722“ “1723“ “2508“ ”9602“ “9603“ 9604“ “9606“ “9607“

^b^We restricted to final 9^th^ grade exams (variables BEDOEMMELSESFORM = “afgangsprøve” and GRUNDSKOLENIVEAU = “FP9”) and used the grade on the 7-point scale (variable GRUNDSKOLEKARAKTER)

^c^DREAM data are recorded since July 1991 and therefore analyses of these outcomes were restricted to those with baseline in August 1991 or later.

^d^Using information at 1 year before the child was born or, if missing, the earliest year education information is available up until baseline at age 18 years of the child).

# **Table S2. Comparison of the percentile of the income distribution at age 30 years in children with atopic dermatitis compared with children without atopic dermatitis (main analysis) and siblings without atopic dermatitis (secondary analysis)**

| **Analysis** | **With atopic dermatitis** | | **Without atopic dermatitis** | | **Mean percentile difference (95% CI)** | | |
| --- | --- | --- | --- | --- | --- | --- | --- |
|  | **No. at risk** | **Mean percentile (SD)** | **No. at risk** | **Mean percentile (SD)** | **Unadjusted^a^** | **Comorbidity-adjusted^b^** | **Fully adjusted^c^** |
| Main analysis | 8409 | 48.8 (29.5) | 853228 | 51.0 (28.9) | -2.1 (-2.7--1.5) | -1.4 (-2.0--0.7) | -1.2 (-1.8--0.6) |
| Sibling analysis | 5119 | 50.2 (29.3) | 6352 | 50.8 (29.0) | -0.6 (-1.7-0.5) | -0.3 (-1.4-0.8) | -0.4 (-1.5-0.8) |

SD standard deviation

^a^Unadjusted model but conditioned on family in the sibling comparison. Adjust indirectly for the calendar year and sex, which are integrated into the calculation of the percentile of the income.

^b^Adjusting also for epilepsy, asthma, rhinitis, any non-psychiatric comorbidity, and birth order (in sibling analyses)

^c^Adjusting also for maternal income and education level

# **Table S3. Number of events, persons at risk, prevalence, and relative risks with 95% confidence intervals (CIs) of various socioeconomic outcomes by age 30 in children with atopic dermatitis compared with children without atopic dermatitis (main analysis) and siblings without atopic dermatitis (secondary analysis)**

| **Analysis, Outcome** | **With atopic dermatitis** | | **Without atopic dermatitis** | | **Relative risk (95% confidence interval)** | | | |
| --- | --- | --- | --- | --- | --- | --- | --- | --- |
|  | **No. of events** | **Prevalence, %** | **No. of events** | **Prevalence, %** | **Unadjusted^a^** | **Minimally-adjusted^b^** | **Comorbidity-adjusted^c^** | **Fully adjusted^d^** |
| **Main analysis** |  |  |  |  |  |  |  |  |
| Earned income in lowest quartile | 2,298 | 27.3 | 206,361 | 24.2 | 1.13 (1.09-1.17) | 1.13 (1.09-1.17) | 1.08 (1.04-1.12) | 1.07 (1.04-1.11) |
| Economic self-sufficient | 8,348 | 99.3 | 848,269 | 99.4 | 1.00 (1.00-1.00) | 1.00 (1.00-1.00) | 1.00 (1.00-1.00) | 1.00 (1.00-1.00) |
| Long-term unemployment | 1,220 | 14.5 | 115,679 | 13.6 | 1.07 (1.02-1.13) | 1.13 (1.08-1.19) | 1.12 (1.06-1.18) | 1.11 (1.05-1.17) |
| Unemployment, not health-related | 5,871 | 70.7 | 566,223 | 68.5 | 1.03 (1.02-1.05) | 1.03 (1.02-1.05) | 1.02 (1.01-1.04) | 1.02 (1.01-1.03) |
| Health-related work absenteeism | 4,514 | 54.4 | 424,432 | 51.3 | 1.06 (1.04-1.08) | 1.07 (1.05-1.09) | 1.03 (1.01-1.05) | 1.02 (1.00-1.04) |
| Sickness benefits | 4,051 | 48.8 | 394,458 | 47.7 | 1.02 (1.00-1.05) | 1.03 (1.01-1.06) | 1.01 (0.98-1.03) | 1.01 (0.98-1.03) |
| Disability pension | 259 | 3.1 | 16,599 | 2.0 | 1.55 (1.38-1.75) | 1.52 (1.35-1.71) | 1.17 (1.04-1.31) | 1.15 (1.02-1.30) |
| Single partnership status | 1,859 | 22.1 | 165,452 | 19.4 | 1.14 (1.09-1.19) | 1.12 (1.08-1.17) | 1.11 (1.07-1.15) | 1.11 (1.06-1.15) |
| Childlessness | 4,855 | 57.7 | 483,620 | 56.7 | 1.02 (1.00-1.04) | 1.01 (0.99-1.03) | 1.01 (1.00-1.03) | 1.01 (1.00-1.03) |
| Assisted reproduction | 235 | 3.8 | 23,021 | 3.3 | 1.14 (1.00-1.29) | 1.05 (0.92-1.19) | 1.01 (0.89-1.14) | 1.01 (0.89-1.15) |
| **Sibling analysis** |  |  |  |  |  |  |  |  |
| Earned income in lowest quartile | 1,305 | 25.5 | 1,552 | 24.4 | 1.04 (0.98-1.11) | 1.05 (0.98-1.11) | 1.02 (0.95-1.09) | 1.02 (0.95-1.09) |
| Economic self-sufficient | 5,090 | 99.4 | 6,315 | 99.4 | 1.00 (1.00-1.00) | 1.00 (1.00-1.00) | 1.00 (1.00-1.00) | 1.00 (1.00-1.00) |
| Long-term unemployment | 706 | 13.8 | 816 | 12.8 | 1.07 (0.98-1.17) | 1.09 (0.99-1.19) | 1.09 (0.99-1.20) | 1.09 (0.99-1.20) |
| Unemployment, not health-related | 3,493 | 68.9 | 4,246 | 67.9 | 1.01 (0.99-1.04) | 1.02 (0.99-1.04) | 1.01 (0.99-1.04) | 1.01 (0.99-1.04) |
| Health-related work absenteeism | 2,734 | 53.9 | 3,237 | 51.8 | 1.04 (1.01-1.08) | 1.05 (1.02-1.08) | 1.03 (1.00-1.07) | 1.03 (0.99-1.07) |
| Sickness benefits | 2,489 | 49.1 | 2,975 | 47.6 | 1.03 (1.00-1.07) | 1.04 (1.00-1.08) | 1.03 (0.99-1.07) | 1.02 (0.98-1.06) |
| Disability pension | 135 | 2.7 | 144 | 2.3 | 1.16 (0.92-1.45) | 1.15 (0.92-1.44) | 1.09 (0.87-1.37) | 1.11 (0.88-1.39) |
| Single partnership status | 1,109 | 21.7 | 1,230 | 19.4 | 1.12 (1.04-1.20) | 1.10 (1.03-1.18) | 1.07 (0.99-1.15) | 1.07 (0.99-1.15) |
| Childlessness | 2,925 | 57.1 | 3,559 | 56.0 | 1.02 (0.99-1.05) | 1.01 (0.98-1.04) | 1.00 (0.97-1.03) | 1.00 (0.97-1.03) |
| Assisted reproduction | 174 | 4.2 | 206 | 4.0 | 1.06 (0.87-1.30) | 1.04 (0.86-1.27) | 0.97 (0.79-1.20) | 0.96 (0.78-1.19) |

^a^Unadjusted model but conditioned on family in the sibling comparison

^b^Adjusting for sex and calendar year at baseline

^c^Adjusting also for epilepsy, asthma, rhinitis, any non-psychiatric comorbidity, and birth order (in sibling analyses)

^d^Adjusting also for maternal income and education level

#

# **Table S4. Association between atopic dermatitis (AD) and percentile of the income distribution at age 30 according to subgroups of age at AD diagnosis, sex, AD severity, AD activity, hand or contact dermatitis, and maternal socioeconomic position**

| **Subgroup** | **Atopic dermatitis** | | **Without atopic dermatitis** | | **Mean percentile difference (95% CI)** | |
| --- | --- | --- | --- | --- | --- | --- |
|  | **No. at risk** | **Mean percentile (SD)** | **No. at risk** | **Mean percentile (SD)** | **Unadjusted^a^** | **Fully-adjusted^ab^** |
| Overall | 8,409 | 48.8 (29.5) | 853,228 | 51.0 (28.9) | -2.1 (-2.7--1.5) | -1.2 (-1.8--0.6) |
| Age at AD diagnosis: 0-4 years | 4,758 | 47.6 (29.4) | 853,228 | 51.0 (28.9) | -3.4 (-4.2--2.6) | -2.0 (-2.8--1.2) |
| Age at AD diagnosis: 5+ years | 3,651 | 50.5 (29.4) | 853,228 | 51.0 (28.9) | -0.5 (-1.5-0.5) | -0.2 (-1.2-0.8) |
| Sex: Female | 3,899 | 49.2 (29.4) | 413,363 | 51.1 (28.9) | -1.9 (-2.8--0.9) | -0.9 (-1.8-0.0) |
| Sex: Male | 4,510 | 48.5 (29.5) | 439,865 | 50.9 (28.9) | -2.3 (-3.2--1.4) | -1.5 (-2.4--0.6) |
| AD severity: moderate | 5,620 | 48.6 (29.4) | 470,300 | 50.6 (28.8) | -2.0 (-2.8--1.2) | -1.2 (-2.0--0.4) |
| AD severity: severe | 127 | 47.8 (30.1) | 470,300 | 50.6 (28.8) | -2.8 (-8.0-2.7) | -1.7 (-6.7-3.6) |
| AD activity: active | 1,150 | 47.3 (29.1) | 470,300 | 50.6 (28.8) | -3.3 (-5.1--1.6) | -2.7 (-4.4--0.9) |
| AD activity: not-active | 4,597 | 48.9 (29.5) | 470,300 | 50.6 (28.8) | -1.7 (-2.5--0.8) | -0.9 (-1.7--0.0) |
| Hand/contact dermatitis: yes | 270 | 46.1 (29.7) | 853,228 | 51.0 (28.9) | -4.8 (-8.6--1.6) | -4.0 (-7.7--0.8) |
| Hand/contact dermatitis: no | 8,139 | 48.9 (29.5) | 853,228 | 51.0 (28.9) | -2.0 (-2.7--1.4) | -1.1 (-1.8--0.5) |
| Maternal income: low | 1,365 | 43.4 (29.3) | 142,995 | 47.0 (29.2) | -3.6 (-5.1--2.2) | -1.7 (-3.2--0.2) |
| Maternal income: medium | 4,927 | 49.3 (29.5) | 510,391 | 51.2 (28.8) | -1.9 (-2.7--1.1) | -1.0 (-1.7--0.1) |
| Maternal income: high | 2,117 | 51.2 (29.1) | 199,842 | 53.1 (28.7) | -1.9 (-3.1--0.6) | -1.4 (-2.6--0.1) |
| Maternal educational level: lower secondary | 2,544 | 41.9 (27.9) | 224,655 | 45.2 (27.5) | -3.4 (-4.4--2.3) | -2.3 (-3.3--1.2) |
| Maternal educational level: upper secondary | 3,661 | 51.8 (29.1) | 401,866 | 52.7 (28.6) | -0.9 (-1.8-0.1) | -0.4 (-1.3-0.6) |
| Maternal educational level: higher | 2,204 | 52.0 (30.6) | 226,707 | 53.6 (30.1) | -1.6 (-2.9--0.3) | -1.4 (-2.7--0.2) |

^a^Adjust indirectly for the calendar year and sex, which are integrated into the calculation of the percentile of the income.

**^b^**Adjusted additionally for epilepsy, asthma, rhinitis, any non-psychiatric comorbidity, and maternal income and education level

# **Table S5. Association between atopic dermatitis (AD) and long-term unemployment, single partnership status and childlessness by age 30 according to subgroups of age at AD diagnosis, sex, AD severity, AD activity, hand or contact dermatitis, and mother’s socioeconomic position**

| **Outcome and subgroup** | **Atopic dermatitis** | | **Without atopic dermatitis** | | **Risk ratio (95% CI)** | |
| --- | --- | --- | --- | --- | --- | --- |
|  | **No. of events** | **Prevalence, %** | **No. of events** | **Prevalence, %** | **Unadjusted** | **Fully adjusted^a^** |
| **Long-term unemployment** | 1,220 | 14.5 | 115,679 | 13.6 | 1.07 (1.02-1.13) | 1.11 (1.05-1.17) |
| Age at AD diagnosis: 0-4 years | 713 | 15.0 | 115,679 | 13.6 | 1.11 (1.03-1.18) | 1.13 (1.05-1.20) |
| Age at AD diagnosis: 5+ years | 507 | 13.9 | 115,679 | 13.6 | 1.02 (0.94-1.11) | 1.08 (1.00-1.18) |
| Sex: Female | 543 | 13.9 | 56,584 | 13.7 | 1.02 (0.94-1.10) | 1.09 (1.01-1.18) |
| Sex: Male | 677 | 15.0 | 59,095 | 13.4 | 1.12 (1.04-1.20) | 1.12 (1.05-1.21) |
| AD severity: moderate | 738 | 13.1 | 55,063 | 11.7 | 1.12 (1.05-1.20) | 1.10 (1.02-1.17) |
| AD severity: severe | 17 | 13.4 | 55,063 | 11.7 | 1.14 (0.73-1.78) | 1.12 (0.72-1.74) |
| AD activity: active | 145 | 12.6 | 55,063 | 11.7 | 1.08 (0.92-1.25) | 1.07 (0.92-1.25) |
| AD activity: not-active | 610 | 13.3 | 55,063 | 11.7 | 1.13 (1.05-1.22) | 1.10 (1.02-1.19) |
| Hand/contact dermatitis: yes | 34 | 12.6 | 115,679 | 13.6 | 0.93 (0.68-1.27) | 0.97 (0.71-1.33) |
| Hand/contact dermatitis: no | 1,186 | 14.6 | 115,679 | 13.6 | 1.07 (1.02-1.13) | 1.11 (1.05-1.17) |
| Maternal income: low | 261 | 19.1 | 24,053 | 16.8 | 1.14 (1.02-1.27) | 1.17 (1.05-1.30) |
| Maternal income: medium | 703 | 14.3 | 69,470 | 13.6 | 1.05 (0.98-1.12) | 1.09 (1.01-1.16) |
| Maternal income: high | 256 | 12.1 | 22,156 | 11.1 | 1.09 (0.97-1.22) | 1.11 (0.99-1.24) |
| Maternal educational level: lower secondary | 464 | 18.2 | 38,542 | 17.2 | 1.06 (0.98-1.15) | 1.11 (1.02-1.21) |
| Maternal educational level: upper secondary | 494 | 13.5 | 52,021 | 12.9 | 1.04 (0.96-1.13) | 1.09 (1.00-1.19) |
| Maternal educational level: higher | 262 | 11.9 | 25,116 | 11.1 | 1.07 (0.96-1.20) | 1.13 (1.01-1.27) |
| **Single partnership status** | 1,859 | 22.1 | 165,452 | 19.4 | 1.14 (1.09-1.19) | 1.11 (1.06-1.15) |
| Age at AD diagnosis: 0-4 years | 1,093 | 23.0 | 165,452 | 19.4 | 1.18 (1.12-1.25) | 1.11 (1.05-1.17) |
| Age at AD diagnosis: 5+ years | 766 | 21.0 | 165,452 | 19.4 | 1.08 (1.02-1.15) | 1.11 (1.04-1.18) |
| Sex: Female | 582 | 14.9 | 58,459 | 14.1 | 1.06 (0.98-1.14) | 1.03 (0.96-1.11) |
| Sex: Male | 1,277 | 28.3 | 106,993 | 24.3 | 1.16 (1.11-1.22) | 1.14 (1.09-1.19) |
| AD severity: moderate | 1,253 | 22.3 | 93,015 | 19.8 | 1.13 (1.07-1.18) | 1.10 (1.05-1.15) |
| AD severity: severe | 33 | 26.0 | 93,015 | 19.8 | 1.31 (0.98-1.76) | 1.35 (1.01-1.81) |
| AD activity: active | 243 | 21.1 | 93,015 | 19.8 | 1.07 (0.96-1.19) | 1.09 (0.98-1.22) |
| AD activity: not-active | 1,043 | 22.7 | 93,015 | 19.8 | 1.15 (1.09-1.21) | 1.11 (1.05-1.17) |
| Hand/contact dermatitis: yes | 75 | 27.8 | 165,452 | 19.4 | 1.43 (1.18-1.74) | 1.41 (1.17-1.71) |
| Hand/contact dermatitis: no | 1,784 | 21.9 | 165,452 | 19.4 | 1.13 (1.08-1.18) | 1.10 (1.05-1.14) |
| Maternal income: low | 343 | 25.1 | 30,841 | 21.6 | 1.17 (1.06-1.28) | 1.10 (1.00-1.21) |
| Maternal income: medium | 1,090 | 22.1 | 99,733 | 19.5 | 1.13 (1.07-1.19) | 1.10 (1.05-1.16) |
| Maternal income: high | 426 | 20.1 | 34,878 | 17.5 | 1.15 (1.06-1.26) | 1.12 (1.03-1.22) |
| Maternal educational level: lower secondary | 574 | 22.6 | 40,377 | 18.0 | 1.26 (1.17-1.35) | 1.18 (1.09-1.26) |
| Maternal educational level: upper secondary | 777 | 21.2 | 76,632 | 19.1 | 1.11 (1.05-1.19) | 1.09 (1.03-1.16) |
| Maternal educational level: higher | 508 | 23.0 | 48,443 | 21.4 | 1.08 (1.00-1.16) | 1.06 (0.98-1.14) |
| **Childlessness** | 4,855 | 57.7 | 483,620 | 56.7 | 1.02 (1.00-1.04) | 1.01 (1.00-1.03) |
| Age at AD diagnosis: 0-4 years | 2,769 | 58.2 | 483,620 | 56.7 | 1.03 (1.00-1.05) | 1.01 (0.99-1.03) |
| Age at AD diagnosis: 5+ years | 2,086 | 57.1 | 483,620 | 56.7 | 1.01 (0.98-1.04) | 1.02 (0.99-1.05) |
| Sex: Female | 1,874 | 48.1 | 199,845 | 48.3 | 0.99 (0.96-1.03) | 1.00 (0.97-1.03) |
| Sex: Male | 2,981 | 66.1 | 283,775 | 64.5 | 1.02 (1.00-1.05) | 1.02 (1.00-1.04) |
| AD severity: moderate | 3,269 | 58.2 | 268,871 | 57.2 | 1.02 (0.99-1.04) | 1.02 (0.99-1.04) |
| AD severity: severe | 80 | 63.0 | 268,871 | 57.2 | 1.10 (0.96-1.26) | 1.14 (1.00-1.30) |
| AD activity: active | 655 | 57.0 | 268,871 | 57.2 | 1.00 (0.95-1.05) | 1.02 (0.97-1.07) |
| AD activity: not-active | 2,694 | 58.6 | 268,871 | 57.2 | 1.03 (1.00-1.05) | 1.02 (1.00-1.04) |
| Hand/contact dermatitis: yes | 158 | 58.5 | 483,620 | 56.7 | 1.03 (0.93-1.14) | 1.04 (0.94-1.15) |
| Hand/contact dermatitis: no | 4,697 | 57.7 | 483,620 | 56.7 | 1.02 (1.00-1.04) | 1.01 (1.00-1.03) |
| Maternal income: low | 780 | 57.1 | 81,012 | 56.7 | 1.01 (0.96-1.06) | 1.00 (0.96-1.05) |
| Maternal income: medium | 2,935 | 59.6 | 295,208 | 57.8 | 1.03 (1.01-1.05) | 1.03 (1.00-1.05) |
| Maternal income: high | 1,140 | 53.8 | 107,400 | 53.7 | 1.00 (0.96-1.04) | 1.00 (0.96-1.04) |
| Maternal educational level: lower secondary | 1,298 | 51.0 | 109,567 | 48.8 | 1.05 (1.01-1.09) | 1.02 (0.99-1.06) |
| Maternal educational level: upper secondary | 2,155 | 58.9 | 228,185 | 56.8 | 1.04 (1.01-1.07) | 1.03 (1.00-1.06) |
| Maternal educational level: higher | 1,402 | 63.6 | 145,868 | 64.3 | 0.99 (0.96-1.02) | 0.99 (0.95-1.02) |

^a^Adjusted for sex, calendar year at baseline, epilepsy, asthma, rhinitis, any non-psychiatric comorbidity, and the mother’s income and education level

# **Table S6. Distribution of variables used for sensitivity analyses**

|  | **Main analysis** | | **Sibling analysis** | |
| --- | --- | --- | --- | --- |
|  | **With AD, no. (%)** | **Without AD, no. (%)** | **With AD, no. (%)** | **Without AD, no. (%)** |
| **ADHD, depression, or anxiety disorder** | 159 (1.9) | 8,705 (1.0) | 84 (1.6) | 75 (1.2) |
| **Maternal socioeconomic position, composite score** |  |  |  |  |
| Low | 980 (11.7) | 81,963 (9.6) | 555 (10.8) | 734 (11.6) |
| Medium | 4,618 (54.9) | 489,237 (57.3) | 2,741 (53.5) | 3,403 (53.6) |
| High | 2,811 (33.4) | 282,028 (33.1) | 1,823 (35.6) | 2,215 (34.9) |
| **5-min Apgar score <7 or intrauterine/birth asphyxia** |  |  |  |  |
| Missing/unknown | 1,335 (15.9) | 221,358 (25.9) | 731 (14.3) | 1,114 (17.5) |
| No | 6,575 (78.2) | 596,977 (70.0) | 4,136 (80.8) | 4,962 (78.1) |
| Yes | 499 (5.9) | 34,893 (4.1) | 252 (4.9) | 276 (4.3) |
| **Preterm birth (<37 gestational weeks)** |  |  |  |  |
| Missing/unknown | 1,623 (19.3) | 256,987 (30.1) | 933 (18.2) | 1,382 (21.8) |
| No | 6,444 (76.6) | 568,510 (66.6) | 4,001 (78.2) | 4,773 (75.1) |
| Yes | 342 (4.1) | 27,731 (3.3) | 185 (3.6) | 197 (3.1) |
| **Low birth weight (<2500 g)** |  |  |  |  |
| Missing/unknown | 13 (0.2) | 1,554 (0.2) | 5 (0.1) | 6 (0.1) |
| No | 7,966 (94.7) | 809,888 (94.9) | 4,861 (95.0) | 6,076 (95.7) |
| Yes | 430 (5.1) | 41,786 (4.9) | 253 (4.9) | 270 (4.3) |
| **Parental income level** |  |  |  |  |
| Low (<Q1) | 457 (5.4) | 46,294 (5.4) | 246 (4.8) | 335 (5.3) |
| Moderate (Q1–Q3) | 4,452 (52.9) | 471,688 (55.3) | 2,799 (61.6) | 3,440 (54.2) |
| High (>Q3) | 3,500 (41.6) | 335,246 (39.3) | 2,074 (23.2) | 2,577 (40.6) |
| **Parental highest education level** |  |  |  |  |
| Lower secondary education | 1,262 (15.0) | 104,621 (12.3) | 675 (13.2) | 855 (13.5) |
| Upper secondary education | 4,082 (48.5) | 436,308 (51.1) | 2,387 (46.6) | 2,928 (46.1) |
| Higher education | 3,065 (36.4) | 312,299 (36.6) | 2,057 (40.2) | 2,569 (40.4) |

Abbreviations: ADHD: Attention deficit hyperactivity disorder

# **Table S7. Sensitivity analyses for the association between atopic dermatitis (AD) and percentile of the income distribution at the age of 30.**

| **Subgroup** | **Atopic dermatitis** | | **Without Atopic dermatitis** | | **Mean percentile difference (95% CI)** | |
| --- | --- | --- | --- | --- | --- | --- |
|  | **No. at risk** | **Mean percentile (SD)** | **No. at risk** | **Mean percentile (SD)** | **Unadjusted^a^** | **Adjusted^b^** |
| **Main analysis** | **8,409** | **48.8 (29.5)** | **853,228** | **51.0 (28.9)** | **-2.1 (-2.7--1.5)** | **-1.2 (-1.8--0.6)** |
| Outcome determined at age 35 | 5,009 | 49.7 (29.6) | 592,051 | 51.7 (29.0) | -2.0 (-2.9--1.2) | -1.1 (-2.0--0.3) |
| Outcome determined at age 40 | 2,661 | 50.3 (29.8) | 382,775 | 52.2 (29.0) | -1.9 (-3.0--0.8) | -0.8 (-2.0-0.3) |
| Excluding those with any year of missing data on outcome | 7,521 | 49.2 (29.2) | 759,270 | 51.7 (28.6) | -2.4 (-3.1--1.8) | -1.4 (-2.0--0.7) |
| Using peak income by age 30 instead of income *at* age 30 | 8,409 | 48.6 (29.9) | 853,228 | 50.9 (29.0) | -2.3 (-2.9--1.6) | -1.3 (-1.9--0.7) |
| Adjusting for ADD/ADHD, depression, and anxiety disorder | 8,409 | 48.8 (29.5) | 853,228 | 51.0 (28.9) |  | -1.2 (-1.8--0.6) |
| Adjusting for composite score maternal socioeconomic position | 8,409 | 48.8 (29.5) | 853,228 | 51.0 (28.9) |  | -1.2 (-1.9--0.6) |
| Reclassifying missing maternal education level as no education | 10,202 | 47.6 (29.3) | 1,066,484 | 50.0 (28.8) | -2.4 (-3.0--1.8) | -1.5 (-2.1--1.0) |
| Excluding those with adverse birth outcomes | 6,270 | 49.4 (29.4) | 570,053 | 51.1 (28.8) | -1.7 (-2.4--1.0) | -1.0 (-1.7--0.3) |
| Excluding patients who did not contribute to the sibling analysis | 5,119 | 50.2 (29.3) | 853,228 | 51.0 (28.9) | -0.8 (-1.5-0.1) | -0.1 (-0.8-0.8) |
| Restricted to those with baseline date in 1996 or later | 7,104 | 48.9 (29.5) | 635,440 | 50.8 (28.8) | -1.9 (-2.6--1.1) | -1.1 (-1.7--0.3) |
| Excluding unexposed without prescriptions for topical AD drugs | 7,104 | 48.9 (29.5) | 436,901 | 50.8 (28.9) | -2.0 (-2.6--1.3) | -1.1 (-1.8--0.4) |
| Adjusting for highest of parental income and education | 8,409 | 48.8 (29.5) | 853,228 | 51.0 (28.9) |  | -1.2 (-1.8--0.6)^d^ |

^a^Adjust indirectly for the calendar year and sex, which are integrated into the calculation of the percentile of the income.

^b^Adjusted for sex and calendar year (by design), and epilepsy, asthma, rhinitis, any non-psychiatric comorbidity, and maternal income and education level

^b^Adjusted for sex, calendar year at baseline, epilepsy, asthma, rhinitis, any non-psychiatric comorbidity, maternal income and education level, and attention deficit disorder, depression, and anxiety disorder

^c^Adjusted for sex, calendar year at baseline, epilepsy, asthma, rhinitis, any non-psychiatric comorbidity, and the composite score of maternal socioeconomic position (low, moderate, high)

^d^Adjusted for sex, calendar year at baseline, epilepsy, asthma, rhinitis, any non-psychiatric comorbidity, the parental income and education level

# **Table S8. Sensitivity analyses for the association between atopic dermatitis and various socioeconomic outcomes by age 30.**

| **Outcome, analysis** | **Atopic dermatitis** | | **Without atopic dermatitis** | | **Risk ratio (95% confidence interval)** | |
| --- | --- | --- | --- | --- | --- | --- |
|  | **No. of events** | **Prevalence, %** | **No. of events** | **Prevalence, %** | **Unadjusted** | **Adjusted^a^** |
| **Earned income in lowest quartile** | **2,298** | **27.3** | **206,361** | **24.2** | **1.13 (1.09-1.17)** | **1.07 (1.04-1.11)** |
| Outcome determined at age 35 | 1,363 | 27.2 | 139,047 | 23.5 | 1.16 (1.11-1.21) | 1.09 (1.04-1.14) |
| Outcome determined at age 40 | 720 | 27.1 | 88,602 | 23.1 | 1.17 (1.10-1.24) | 1.08 (1.01-1.15) |
| Excluding those with any year of missing data on outcome | 1,985 | 26.4 | 172,901 | 22.8 | 1.16 (1.12-1.20) | 1.09 (1.05-1.13) |
| Adjusting for ADD/ADHD, depression, and anxiety disorder | 2,298 | 27.3 | 206,361 | 24.2 | – | 1.07 (1.03-1.11)^b^ |
| Adjusting for composite score maternal socioeconomic position | 2,298 | 27.3 | 206,361 | 24.2 | – | 1.08 (1.04-1.11)^c^ |
| Reclassifying missing maternal education level as no education | 2,916 | 28.6 | 266,132 | 25.0 | 1.15 (1.11-1.18) | 1.09 (1.05-1.12) |
| Excluding those with adverse birth outcomes | 1,658 | 26.4 | 135,424 | 23.8 | 1.11 (1.07-1.16) | 1.06 (1.02-1.11) |
| Excluding patients who did not contribute to the sibling analysis | 1,305 | 25.5 | 206,361 | 24.2 | 1.05 (1.01-1.10) | 1.01 (0.96-1.05) |
| Restricted to those with baseline date in 1996 or later | 1,937 | 27.3 | 153,930 | 24.2 | 1.13 (1.08-1.17) | 1.07 (1.03-1.11) |
| Excluding unexposed without prescriptions for topical AD drugs | 1,937 | 27.3 | 105,639 | 24.2 | 1.13 (1.09-1.17) | 1.08 (1.04-1.12) |
| Adjusting for highest of parental income and education | 2,298 | 27.3 | 206,361 | 24.2 | – | 1.07 (1.03-1.11)^d^ |
| **Economic self-sufficient** | **8,348** | **99.3** | **848,269** | **99.4** | **1.00 (1.00-1.00)** | **1.00 (1.00-1.00)** |
| Outcome determined at age 35 | 4,989 | 99.6 | 590,104 | 99.6 | 1.00 (1.00-1.00) | 1.00 (1.00-1.00) |
| Outcome determined at age 40 | 2,650 | 99.5 | 381,527 | 99.6 | 1.00 (1.00-1.00) | 1.00 (1.00-1.00) |
| Excluding those with any year of missing data on outcome | 8,136 | 99.5 | 829,736 | 99.7 | 1.00 (1.00-1.00) | 1.00 (1.00-1.00) |
| Adjusting for ADD/ADHD, depression, and anxiety disorder | 8,348 | 99.3 | 848,269 | 99.4 | – | 1.00 (1.00-1.00)^b^ |
| Adjusting for composite score maternal socioeconomic position | 8,348 | 99.3 | 848,269 | 99.4 | – | 1.00 (1.00-1.00)^c^ |
| Reclassifying missing maternal education level as no education | 10,131 | 99.3 | 1,060,376 | 99.4 | 1.00 (1.00-1.00) | 1.00 (1.00-1.00) |
| Excluding those with adverse birth outcomes | 6,225 | 99.3 | 566,762 | 99.4 | 1.00 (1.00-1.00) | 1.00 (1.00-1.00) |
| Excluding patients who did not contribute to the sibling analysis | 5,090 | 99.4 | 848,269 | 99.4 | 1.00 (1.00-1.00) | 1.00 (1.00-1.00) |
| Restricted to those with baseline date in 1996 or later | 7,054 | 99.3 | 631,737 | 99.4 | 1.00 (1.00-1.00) | 1.00 (1.00-1.00) |
| Excluding unexposed without prescriptions for topical AD drugs | 7,054 | 99.3 | 434,257 | 99.4 | 1.00 (1.00-1.00) | 1.00 (1.00-1.00) |
| Adjusting for highest of parental income and education | 8,348 | 99.3 | 848,269 | 99.4 | – | 1.00 (1.00-1.00)^d^ |
| **Long-term unemployment** | **1,220** | **14.5** | **115,679** | **13.6** | **1.07 (1.02-1.13)** | **1.11 (1.05-1.17)** |
| Outcome determined at age 35 | 1,038 | 20.7 | 111,143 | 18.8 | 1.10 (1.05-1.17) | 1.14 (1.08-1.21) |
| Outcome determined at age 40 | 682 | 25.6 | 87,932 | 23.0 | 1.12 (1.05-1.19) | 1.13 (1.06-1.21) |
| Excluding those with any year of missing data on outcome | 1,204 | 14.7 | 114,296 | 13.7 | 1.07 (1.02-1.13) | 1.11 (1.05-1.17) |
| Adjusting for ADD/ADHD, depression, and anxiety disorder | 1,220 | 14.5 | 115,679 | 13.6 | – | 1.11 (1.05-1.17)^b^ |
| Adjusting for composite score maternal socioeconomic position | 1,220 | 14.5 | 115,679 | 13.6 | – | 1.11 (1.05-1.17)^c^ |
| Reclassifying missing maternal education level as no education | 1,594 | 15.6 | 160,303 | 15.0 | 1.04 (0.99-1.09) | 1.10 (1.05-1.15) |
| Excluding those with adverse birth outcomes | 812 | 13.0 | 65,774 | 11.5 | 1.12 (1.05-1.20) | 1.10 (1.03-1.17) |
| Excluding patients who did not contribute to the sibling analysis | 706 | 13.8 | 115,679 | 13.6 | 1.02 (0.95-1.09) | 1.05 (0.98-1.13) |
| Restricted to those with baseline date in 1996 or later | 938 | 13.2 | 73,941 | 11.6 | 1.13 (1.07-1.20) | 1.10 (1.04-1.17) |
| Excluding unexposed without prescriptions for topical AD drugs | 938 | 13.2 | 50,467 | 11.6 | 1.14 (1.08-1.21) | 1.11 (1.05-1.18) |
| Adjusting for highest of parental income and education | 1,220 | 14.5 | 115,679 | 13.6 | – | 1.11 (1.05-1.17)^d^ |
| **Unemployment, not health-related** | **5,871** | **70.7** | **566,223** | **68.5** | **1.03 (1.02-1.05)** | **1.02 (1.01-1.03)** |
| Outcome determined at age 35 | 3,653 | 74.5 | 408,837 | 72.3 | 1.03 (1.01-1.05) | 1.03 (1.02-1.05) |
| Outcome determined at age 40 | 1,985 | 77.8 | 268,104 | 75.2 | 1.03 (1.01-1.06) | 1.03 (1.01-1.06) |
| Excluding those with any year of missing data on outcome | NA | NA | NA | NA | NA | NA |
| Adjusting for ADD/ADHD, depression, and anxiety disorder | 5,871 | 69.8 | 566,223 | 66.4 | – | 1.02 (1.01-1.03)^b^ |
| Adjusting for composite score maternal socioeconomic position | 5,871 | 69.8 | 566,223 | 66.4 | – | 1.02 (1.01-1.04)^c^ |
| Reclassifying missing maternal education level as no education | 7,205 | 71.7 | 713,746 | 69.6 | 1.03 (1.02-1.04) | 1.02 (1.01-1.03) |
| Excluding those with adverse birth outcomes | 4,394 | 70.1 | 383,762 | 67.3 | 1.04 (1.02-1.06) | 1.02 (1.00-1.04) |
| Excluding patients who did not contribute to the sibling analysis | 3,493 | 68.9 | 566,223 | 68.5 | 1.01 (0.99-1.02) | 1.00 (0.98-1.02) |
| Restricted to those with baseline date in 1996 or later | 4,975 | 70.0 | 428,707 | 67.5 | 1.04 (1.02-1.05) | 1.02 (1.00-1.03) |
| Excluding unexposed without prescriptions for topical AD drugs | 4,975 | 70.0 | 290,589 | 66.5 | 1.05 (1.04-1.07) | 1.02 (1.00-1.04) |
| Adjusting for highest of parental income and education | 5,871 | 70.7 | 566,223 | 68.5 |  | 1.02 (1.01-1.04)^d^ |
| **Health-related work absenteeism** | **4,514** | **54.4** | **424,432** | **51.3** | **1.06 (1.04-1.08)** | **1.02 (1.00-1.04)** |
| Outcome determined at age 35 | 3,205 | 65.4 | 349,864 | 61.8 | 1.06 (1.04-1.08) | 1.02 (1.00-1.04) |
| Outcome determined at age 40 | 1,812 | 71.0 | 239,085 | 67.1 | 1.06 (1.03-1.08) | 1.03 (1.01-1.06) |
| Excluding those with any year of missing data on outcome | NA | NA | NA | NA | NA | NA |
| Adjusting for ADD/ADHD, depression, and anxiety disorder | 4,514 | 53.7 | 424,432 | 49.7 | – | 1.02 (1.00-1.04)^b^ |
| Adjusting for composite score maternal socioeconomic position | 4,514 | 53.7 | 424,432 | 49.7 | – | 1.02 (1.00-1.04)^c^ |
| Reclassifying missing maternal education level as no education | 5,646 | 56.2 | 547,616 | 53.4 | 1.05 (1.03-1.07) | 1.02 (1.01-1.04) |
| Excluding those with adverse birth outcomes | 3,383 | 54.0 | 292,051 | 51.2 | 1.05 (1.03-1.08) | 1.02 (1.00-1.05) |
| Excluding patients who did not contribute to the sibling analysis | 2,734 | 53.9 | 424,432 | 51.3 | 1.05 (1.02-1.08) | 1.03 (1.00-1.06) |
| Restricted to those with baseline date in 1996 or later | 3,856 | 54.3 | 328,008 | 51.6 | 1.05 (1.03-1.07) | 1.02 (1.00-1.04) |
| Excluding unexposed without prescriptions for topical AD drugs | 3,856 | 54.3 | 220,904 | 50.6 | 1.07 (1.05-1.10) | 1.04 (1.02-1.06) |
| Adjusting for highest of parental income and education | 4,514 | 54.4 | 424,432 | 51.3 | – | 1.03 (1.01-1.05)^d^ |
| **Sickness benefit** | **4,051** | **48.8** | **394,458** | **47.7** | **1.02 (1.00-1.05)** | **1.01 (0.98-1.03)** |
| Outcome determined at age 35 | 2,962 | 60.4 | 331,283 | 58.6 | 1.03 (1.01-1.06) | 1.01 (0.99-1.03) |
| Outcome determined at age 40 | 1,700 | 66.6 | 229,223 | 64.3 | 1.04 (1.01-1.06) | 1.02 (0.99-1.05) |
| Excluding those with any year of missing data on outcome | NA | NA | NA | NA | NA | NA |
| Adjusting for ADD/ADHD, depression, and anxiety disorder | 4,051 | 48.2 | 394,458 | 46.2 | – | 1.01 (0.98-1.03) ^b^ |
| Adjusting for composite score maternal socioeconomic position | 4,051 | 48.2 | 394,458 | 46.2 | – | 1.00 (0.98-1.03) ^c^ |
| Reclassifying missing maternal education level as no education | 5,029 | 50.1 | 506,282 | 49.4 | 1.01 (0.99-1.03) | 1.00 (0.98-1.02) |
| Excluding those with adverse birth outcomes | 3,062 | 48.8 | 272,832 | 47.9 | 1.02 (0.99-1.05) | 1.00 (0.98-1.03) |
| Excluding patients who did not contribute to the sibling analysis | 2,489 | 49.1 | 394,458 | 47.7 | 1.03 (1.00-1.06) | 1.02 (0.99-1.05) |
| Restricted to those with baseline date in 1996 or later | 3,454 | 48.6 | 303,927 | 47.8 | 1.02 (0.99-1.04) | 1.00 (0.98-1.03) |
| Excluding unexposed without prescriptions for topical AD drugs | 3,454 | 48.6 | 205,125 | 46.9 | 1.04 (1.01-1.06) | 1.02 (1.00-1.05) |
| Adjusting for highest of parental income and education | 4,051 | 48.8 | 394,458 | 47.7 |  | 1.01 (0.99-1.03)^d^ |
| **Disability pension** | **259** | **3.1** | **16,599** | **2.0** | **1.55 (1.38-1.75)** | **1.15 (1.02-1.30)** |
| Outcome determined at age 35 | 195 | 4.0 | 15,335 | 2.7 | 1.47 (1.28-1.69) | 1.11 (0.97-1.28) |
| Outcome determined at age 40 | 127 | 5.0 | 11,947 | 3.4 | 1.48 (1.25-1.76) | 1.12 (0.94-1.33) |
| Excluding those with any year of missing data on outcome | NA | NA | NA | NA | NA | NA |
| Adjusting for ADD/ADHD, depression, and anxiety disorder | 259 | 3.1 | 16,599 | 1.9 | – | 1.14 (1.00-1.29) ^b^ |
| Adjusting for composite score maternal socioeconomic position | 259 | 3.1 | 16,599 | 1.9 | – | 1.17 (1.04-1.32) ^c^ |
| Reclassifying missing maternal education level as no education | 357 | 3.6 | 22,978 | 2.2 | 1.59 (1.43-1.76) | 1.19 (1.07-1.32) |
| Excluding those with adverse birth outcomes | 169 | 2.7 | 10,067 | 1.8 | 1.53 (1.31-1.77) | 1.21 (1.04-1.40) |
| Excluding patients who did not contribute to the sibling analysis | 135 | 2.7 | 16,599 | 2.0 | 1.33 (1.12-1.57) | 1.05 (0.89-1.24) |
| Restricted to those with baseline date in 1996 or later | 225 | 3.2 | 13,472 | 2.1 | 1.49 (1.31-1.70) | 1.14 (1.00-1.30) |
| Excluding unexposed without prescriptions for topical AD drugs | 225 | 3.2 | 8,738 | 2.0 | 1.58 (1.39-1.80) | 1.22 (1.07-1.39) |
| Adjusting for highest of parental income and education | 259 | 3.1 | 16,599 | 2.0 |  | 1.15 (1.03-1.30)^d^ |
| **Single partnership status** | **1,859** | **22.1** | **165,452** | **19.4** | **1.14 (1.09-1.19)** | **1.11 (1.06-1.15)** |
| Outcome determined at age 35 | 668 | 13.3 | 67,695 | 11.4 | 1.17 (1.09-1.25) | 1.11 (1.04-1.20) |
| Outcome determined at age 40 | 283 | 10.6 | 33,336 | 8.7 | 1.22 (1.09-1.36) | 1.15 (1.03-1.29) |
| Excluding those with any year of missing data on outcome | 1,544 | 20.4 | 135,781 | 17.8 | 1.15 (1.10-1.20) | 1.11 (1.06-1.16) |
| Adjusting for ADD/ADHD, depression, and anxiety disorder | 1,859 | 22.1 | 165,452 | 19.4 | – | 1.11 (1.06-1.15) ^b^ |
| Adjusting for composite score maternal socioeconomic position | 1,859 | 22.1 | 165,452 | 19.4 | – | 1.11 (1.07-1.15) ^c^ |
| Reclassifying missing maternal education level as no education | 2,256 | 22.1 | 207,511 | 19.5 | 1.14 (1.10-1.18) | 1.10 (1.06-1.14) |
| Excluding those with adverse birth outcomes | 1,357 | 21.6 | 108,498 | 19.0 | 1.14 (1.08-1.19) | 1.11 (1.06-1.17) |
| Excluding patients who did not contribute to the sibling analysis | 1,109 | 21.7 | 165,452 | 19.4 | 1.12 (1.06-1.18) | 1.08 (1.03-1.14) |
| Restricted to those with baseline date in 1996 or later | 1,573 | 22.1 | 124,357 | 19.6 | 1.13 (1.08-1.18) | 1.10 (1.05-1.15) |
| Excluding unexposed without prescriptions for topical AD drugs | 1,573 | 22.1 | 87,738 | 20.1 | 1.10 (1.06-1.15) | 1.10 (1.05-1.15) |
| Adjusting for highest of parental income and education | 1,859 | 22.1 | 165,452 | 19.4 | – | 1.11 (1.06-1.15)^d^ |
| **Childlessness** | **4,855** | **57.7** | **483,620** | **56.7** | **1.02 (1.00-1.04)** | **1.01 (1.00-1.03)** |
| Outcome determined at age 35 | 1,643 | 32.8 | 179,230 | 30.3 | 1.08 (1.04-1.13) | 1.05 (1.01-1.10) |
| Outcome determined at age 40 | 624 | 23.4 | 80,143 | 20.9 | 1.12 (1.05-1.20) | 1.06 (0.99-1.13) |
| Excluding those with any year of missing data on outcome | NA | NA | NA | NA | NA | NA |
| Adjusting for ADD/ADHD, depression, and anxiety disorder | 4,855 | 57.7 | 483,620 | 56.7 | – | 1.01 (1.00-1.03)^b^ |
| Adjusting for composite score maternal socioeconomic position | 4,855 | 57.7 | 483,620 | 56.7 | – | 1.02 (1.00-1.03)^c^ |
| Reclassifying missing maternal education level as no education | 5,824 | 57.1 | 593,033 | 55.6 | 1.03 (1.01-1.04) | 1.02 (1.00-1.04) |
| Excluding those with adverse birth outcomes | 3,594 | 57.3 | 321,686 | 56.4 | 1.02 (0.99-1.04) | 1.01 (0.99-1.03) |
| Excluding still births | 4,934 | 58.7 | 491,495 | 57.6 | 1.02 (1.00-1.04) | 1.01 (0.99-1.03) |
| Excluding patients who did not contribute to the sibling analysis | 2,925 | 57.1 | 483,620 | 56.7 | 1.01 (0.98-1.03) | 1.00 (0.97-1.02) |
| Restricted to those with baseline date in 1996 or later | 4,106 | 57.8 | 360,890 | 56.8 | 1.02 (1.00-1.04) | 1.01 (0.99-1.03) |
| Excluding unexposed without prescriptions for topical AD drugs | 4,106 | 57.8 | 252,260 | 57.7 | 1.00 (0.98-1.02) | 1.01 (0.99-1.03) |
| Adjusting for highest of parental income and education | 4,855 | 57.7 | 483,620 | 56.7 | – | 1.01 (1.00-1.03)^d^ |
| **Assisted reproduction** | **235** | **3.8** | **23,021** | **3.3** | **1.14 (1.00-1.29)** | **1.01 (0.89-1.15)** |
| Outcome determined at age 35 | 266 | 7.8 | 36,861 | 8.0 | 0.98 (0.87-1.10) | 0.94 (0.84-1.06) |
| Outcome determined at age 40 | 182 | 11.1 | 28,305 | 10.9 | 1.02 (0.89-1.17) | 1.01 (0.88-1.17) |
| Excluding those with any year of missing data on outcome | NA | NA | NA | NA | NA | NA |
| Adjusting for ADD/ADHD, depression, and anxiety disorder | 235 | 2.8 | 23,021 | 2.7 | – | 1.01 (0.89-1.15)^b^ |
| Adjusting for composite score maternal socioeconomic position | 235 | 2.8 | 23,021 | 2.7 | – | 1.01 (0.89-1.14)^c^ |
| Reclassifying missing maternal education level as no education | 287 | 3.7 | 28,824 | 3.2 | 1.13 (1.01-1.27) | 1.00 (0.89-1.12) |
| Excluding those with adverse birth outcomes | 191 | 4.5 | 17,447 | 4.1 | 1.08 (0.94-1.24) | 1.03 (0.90-1.19) |
| Excluding patients who did not contribute to the sibling analysis | 174 | 4.2 | 23,021 | 3.3 | 1.26 (1.09-1.46) | 1.10 (0.95-1.28) |
| Restricted to those with baseline date in 1996 or later | 216 | 4.4 | 19,349 | 4.1 | 1.08 (0.95-1.23) | 1.04 (0.91-1.18) |
| Excluding unexposed without prescriptions for topical AD drugs | 216 | 4.4 | 13,505 | 3.9 | 1.14 (1.00-1.30) | 1.05 (0.92-1.20) |
| Adjusting for highest of parental income and education | 235 | 3.8 | 23,021 | 3.3 | – | 1.01 (0.89-1.15^d^ |

ADD/ADHD: Attention deficit disorder/Attention deficit hyperactivity disorder

^a^Adjusted for sex, calendar year at baseline, epilepsy, asthma, rhinitis, any non-psychiatric comorbidity, and maternal income and education level

^b^Adjusted for sex, calendar year at baseline, epilepsy, asthma, rhinitis, any non-psychiatric comorbidity, the maternal income and education level, and attention deficit disorder, depression, and anxiety disorder

^c^Adjusted for sex, calendar year at baseline, epilepsy, asthma, rhinitis, any non-psychiatric comorbidity, and the composite score of maternal socioeconomic position (low, moderate, high)

^d^Adjusted for sex, calendar year at baseline, epilepsy, asthma, rhinitis, any non-psychiatric comorbidity, the parental income and education level

# **Table S9. Sensitivity analyses for the association between atopic dermatitis (AD) and percentile of the income distribution at age 30, sibling comparison**

| **Analysis** | **With atopic dermatitis** | | **Without atopic dermatitis** | | **Mean percentile difference (95% CI)** | |
| --- | --- | --- | --- | --- | --- | --- |
|  | **No. at risk** | **Mean percentile (SD)** | **No. at risk** | **Mean percentile (SD)** | **Unadjusted^a^** | **Fully adjusted^b^** |
| **Main analysis** | **5,119** | **50.2 (29.3)** | **6,352** | **50.8 (29.0)** | **-0.6 (-1.7-0.5)** | **-0.4 (-1.5-0.8)** |
| Restricting to siblings with an age difference of ≤3 years | 2,470 | 49.7 (29.8) | 2,583 | 51.2 (29.6) | -1.5 (-3.2-0.2) | -1.4 (-3.1-0.4) |
| Restricting to siblings without prescriptions for topical AD drugs | 3,116 | 50.0 (29.4) | 3,530 | 50.8 (28.8) | -0.8 (-2.4-0.5) | -0.5 (-2.1-0.9) |

SD standard deviation

^a^Unadjusted model but conditioned on family. Adjust indirectly for the calendar year and sex by the definition of the percentile of income.

^b^Adjusting also for epilepsy, asthma, rhinitis, any non-psychiatric comorbidity, birth order, and the maternal income and education level

# **Table S10. Sensitivity analyses for the association between atopic dermatitis and various socioeconomic outcomes by age 30, sibling comparison**

| **Outcome, analysis** | **Atopic dermatitis** | | **Without atopic dermatitis** | | **Risk ratio (95% confidence interval)** | |
| --- | --- | --- | --- | --- | --- | --- |
|  | **No. of events** | **Prevalence, %** | **No. of events** | **Prevalence, %** | **Unadjusted^a^** | **Adjusted^b^** |
| **Earned income in lowest quartile** | **1,305** | **25.5** | **1,552** | **24.4** | **1.04 (0.98-1.11)** | **1.02 (0.95-1.09)** |
| Restricting to siblings with an age difference of ≤3 years | 658 | 26.6 | 651 | 25.2 | 1.06 (0.97-1.16) | 1.03 (0.94-1.14) |
| Restricting to siblings without prescriptions for topical AD drugs | 817 | 26.2 | 851 | 24.1 | 1.09 (1.00-1.18) | 1.06 (0.98-1.16) |
| **Economic self-sufficient** | **5,090** | **99.4** | **6,315** | **99.4** | **1.00 (1.00-1.00)** | **1.00 (1.00-1.00)** |
| Restricting to siblings with an age difference of ≤3 years | 2,453 | 99.3 | 2,566 | 99.3 | 1.00 (1.00-1.00) | 1.00 (1.00-1.01) |
| Restricting to siblings without prescriptions for topical AD drugs | 3,096 | 99.4 | 3,508 | 99.4 | 1.00 (1.00-1.00) | 1.00 (1.00-1.01) |
| **Long-term unemployment** | **706** | **13.8** | **816** | **12.8** | **1.07 (0.98-1.17)** | **1.09 (0.99-1.20)** |
| Restricting to siblings with an age difference of ≤3 years | 325 | 13.2 | 327 | 12.7 | 1.04 (0.91-1.19) | 1.08 (0.93-1.25) |
| Restricting to siblings without prescriptions for topical AD drugs | 413 | 13.3 | 413 | 11.7 | 1.13 (1.00-1.28) | 1.13 (0.99-1.29) |
| **Unemployment, not health-related** | **3,493** | **68.9** | **4,246** | **67.9** | **1.01 (0.99-1.04)** | **1.01 (0.99-1.04)** |
| Restricting to siblings with an age difference of ≤3 years | 1,714 | 70.0 | 1,718 | 67.5 | 1.04 (1.00-1.07) | 1.04 (1.00-1.08) |
| Restricting to siblings without prescriptions for topical AD drugs | 2,121 | 68.4 | 2,331 | 66.0 | 1.04 (1.00-1.07) | 1.04 (1.00-1.07) |
| **Health-related work absenteeism** | **2,734** | **53.9** | **3,237** | **51.8** | **1.04 (1.01-1.08)** | **1.03 (0.99-1.07)** |
| Restricting to siblings with an age difference of ≤3 years | 1,319 | 53.9 | 1,290 | 50.6 | 1.06 (1.01-1.12) | 1.05 (1.00-1.11) |
| Restricting to siblings without prescriptions for topical AD drugs | 1,678 | 54.1 | 1,776 | 50.3 | 1.08 (1.03-1.12) | 1.05 (1.00-1.10) |
| **Sickness benefit** | **2,489** | **49.1** | **2,975** | **47.6** | **1.03 (1.00-1.07)** | **1.02 (0.98-1.06)** |
| Restricting to siblings with an age difference of ≤3 years | 1,175 | 48.0 | 1,185 | 46.5 | 1.03 (0.98-1.09) | 1.02 (0.96-1.08) |
| Restricting to siblings without prescriptions for topical AD drugs | 1,528 | 49.3 | 1,630 | 46.2 | 1.07 (1.02-1.12) | 1.03 (0.98-1.09) |
| **Disability pension** | **135** | **2.7** | **144** | **2.3** | **1.16 (0.92-1.45)** | **1.11 (0.88-1.39)** |
| Restricting to siblings with an age difference of ≤3 years | 77 | 3.1 | 63 | 2.5 | 1.27 (0.93-1.73) | 1.11 (0.81-1.52) |
| Restricting to siblings without prescriptions for topical AD drugs | 80 | 2.6 | 86 | 2.4 | 1.06 (0.79-1.42) | 1.11 (0.81-1.51) |
| **Single partnership status** | **1,109** | **21.7** | **1,230** | **19.4** | **1.12 (1.04-1.20)** | **1.07 (0.99-1.15)** |
| Restricting to siblings with an age difference of ≤3 years | 539 | 21.8 | 507 | 19.6 | 1.11 (1.00-1.23) | 1.04 (0.93-1.16) |
| Restricting to siblings without prescriptions for topical AD drugs | 662 | 21.2 | 686 | 19.4 | 1.09 (1.00-1.20) | 1.08 (0.98-1.19) |
| **Childlessness** | **2,925** | **57.1** | **3,559** | **56.0** | **1.02 (0.99-1.05)** | **1.00 (0.97-1.03)** |
| Restricting to siblings with an age difference of ≤3 years | 1,430 | 57.9 | 1,406 | 54.4 | 1.06 (1.02-1.11) | 1.04 (0.99-1.10) |
| Restricting to siblings without prescriptions for topical AD drugs | 1,778 | 57.1 | 2,032 | 57.6 | 0.99 (0.95-1.03) | 0.98 (0.94-1.03) |
| **Assisted reproduction** | **174** | **4.2** | **206** | **4.0** | **1.06 (0.87-1.30)** | **0.96 (0.78-1.19)** |
| Restricting to siblings with an age difference of ≤3 years | 81 | 4.2 | 87 | 4.2 | 0.99 (0.74-1.33) | 0.93 (0.68-1.28) |
| Restricting to siblings without prescriptions for topical AD drugs | 103 | 4.2 | 118 | 4.1 | 1.02 (0.79-1.32) | 0.88 (0.67-1.16) |

^a^Unadjusted model conditioning on family

^b^Adjusting for sex, calendar period at baseline, epilepsy, asthma, rhinitis, any non-psychiatric comorbidity, birth order, and maternal income and education level

# **Table S11. *Post hoc* analyses of the distribution of education level and partnership status by the outcomes of partnership and childlessness in those with and without childhood atopic dermatitis (AD)**

1. Distribution of education level by AD and partnership status at age 30

| **Highest education at age 30** | **AD** | | **Without AD** | | |  |
| --- | --- | --- | --- | --- | --- | --- |
|  | **Single until age of 30** | | **Single until age of 30** | | |  |
|  | **Yes, n (%)** | **No, n(%)** | **Yes, n (%)** | **No, n(%)** | |  |
| No education/unknown | 56 (3.0) | 66 (1.0) | 3220 (1.9) | | 5722 (0.8) | |
| Lower secondary | 452 (24.3) | 820 (12.5) | 32,265 (19.5) | | 76,190 (11.1) | |
| Upper secondary | 666 (35.8) | 2,564 (39.1) | 63,738 (38.5) | | 280,238 (40.7) | |
| Higher | 685 (36.8) | 3,100 (47.3) | 66,229 (40.0) | | 325,626 (47.3) | |

1. Distribution of education level by AD and partnership status at age 40

| **Highest education at age 40** | **AD** | | **Without AD** | | |  |
| --- | --- | --- | --- | --- | --- | --- |
|  | **Single until age of 40** | | **Single until age of 40** | | |  |
|  | **Yes, n (%)** | **No, n(%)** | **Yes, n (%)** | **No, n(%)** | |  |
| No education/unknown | 11 (3.9) | 16 (0.7) | 768 (2.3) | | 1763 (0.5) | |
| Lower secondary | 82 (29.0) | 253 (10.6) | 7,781 (23.3) | | 30,209 (8.6) | |
| Upper secondary | 103 (36.4) | 874 (36.7) | 12,865 (38.6) | | 136,353 (39.0) | |
| Higher | 87 (30.7) | 1,236 (52.0) | 11,922 (35.8) | | 181,267 (51.9) | |

1. Distribution of education level by AD and childlessness at age 30

| **Highest education at age 30** | **AD** | | **Without AD** | |
| --- | --- | --- | --- | --- |
|  | **Childlessness at age 30** | | **Childlessness at age 30** | |
|  | **Yes** | **No** | **Yes** | **No** |
|  | **%** | **%** | **%** | **%** |
| No education/unknown | 79 (1.6) | 43 (1.2) | 5046 (1.0) | 3896 (1.1) |
| Lower secondary | 742 (15.3) | 530 (14.9) | 60,957 (12.6) | 47,498 (12.9) |
| Upper secondary | 1,747 (36.0) | 1,483 (41.7) | 180,653 (37.4) | 163,323 (44.2) |
| Higher | 2,287 (47.1) | 1,498 (42.1) | 236,964 (49.0) | 154,891 (41.9) |

1. Distribution of education level by AD and childlessness at age 40

| **Highest education at age 40** | **AD** | | **Without AD** | |
| --- | --- | --- | --- | --- |
|  | **Childlessness at age 40** | | **Childlessness at age 40** | |
|  | **Yes** | **No** | **Yes** | **No** |
|  | **n (%)** | **n (%)** | **n (%)** | **n (%)** |
| No education/unknown | 14 (2.2) | 13 (0.6) | 1117 (1.4) | 1414 (0.5) |
| Lower secondary | 136 (21.8) | 199 (9.8) | 14,329 (17.9) | 23,661 (7.8) |
| Upper secondary | 240 (38.5) | 737 (36.2) | 32,236 (40.2) | 116,982 (38.6) |
| Higher | 234 (37.5) | 1,089 (53.4) | 32,461 (40.5) | 160,728 (53.1) |

1. Distribution of partnership status at age 40 by AD and childlessness at age 40

| **Single until age of 40** | **AD** | | **Without AD** | |
| --- | --- | --- | --- | --- |
|  | **Childlessness at age 40** | | **Childlessness at age 40** | |
|  | **Yes** | **No** | **Yes** | **No** |
|  | **n (%)** | **n (%)** | **n (%)** | **n (%)** |
| No | 364 (58.3) | 2015 (98.9) | 49778 (62.1) | 299814 (99.0) |
| Yes | 260 (41.7) | 23 (1.1) | 30,365 (37.9) | 2,971 (1.0) |

# **Figure S1. Flowchart**


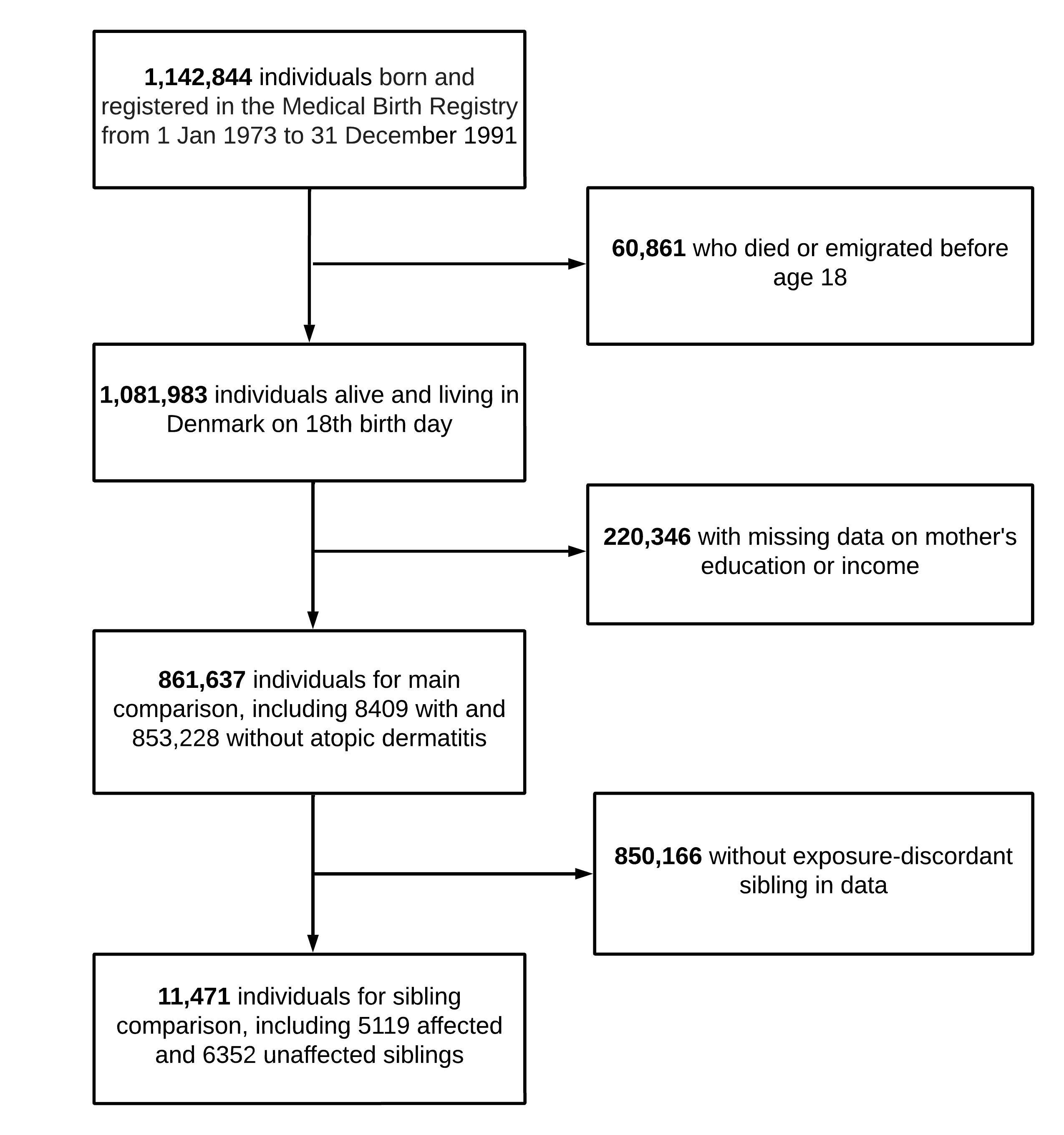


# **References**

1. Schmidt M, Pedersen L, Sørensen HT. The Danish Civil Registration System as a tool in epidemiology. Eur J Epidemiol. 2014;29(8):541-9. doi:10.1007/s10654-014-9930-3

2. Bliddal M, Broe A, Pottegård A, Olsen J, Langhoff-Roos J. The Danish Medical Birth Register. Eur J Epidemiol. 2018;33(1):27-36. doi:10.1007/s10654-018-0356-1

3. Schmidt M, Schmidt SAJ, Sandegaard JL, Ehrenstein V, Pedersen L, Sørensen HT. The Danish National Patient Registry: a review of content, data quality, and research potential. Clin Epidemiol. 2015;7:449-90. doi:10.2147/CLEP.S91125

4. Mors O, Perto GP, Mortensen PB. The Danish Psychiatric Central Research Register. Scand J Public Health. 2011;39(7 Suppl):54-7. doi:10.1177/1403494810395825

5. Pottegård A, Schmidt SAJ, Wallach-Kildemoes H, Sørensen HT, Hallas J, Schmidt M. Data Resource Profile: The Danish National Prescription Registry. Int J Epidemiol. 2017;46(3):798-798f. doi:10.1093/ije/dyw213

6. Blenstrup LT, Knudsen LB. Danish registers on aspects of reproduction. Scand J Public Health. 2011;39(7 Suppl):79-82. doi:10.1177/1403494811399957

7. Jensen VM, Rasmussen AW. Danish Education Registers. Scand J Public Health. 2011;39(7 Suppl):91-4. doi:10.1177/1403494810394715

8. Petersson F, Baadsgaard M, Thygesen LC. Danish registers on personal labour market affiliation. Scand J Public Health. 2011;39(7 Suppl):95-8. doi:10.1177/1403494811408483

9. Hjollund NH, Larsen FB, Andersen JH. Register-based follow-up of social benefits and other transfer payments: accuracy and degree of completeness in a Danish interdepartmental administrative database compared with a population-based survey. Scand J Public Health. 2007;35(5):497-502. doi:10.1080/14034940701271882
